# Two-phase radial endobronchial ultrasound bronchoscopy registration

**DOI:** 10.1117/1.JMI.12.2.025001

**Published:** 2025-03-07

**Authors:** Wennan Zhao, Trevor Kuhlengel, Qi Chang, Vahid Daneshpajooh, Yuxuan He, Austin Kao, Rebecca Bascom, Danish Ahmad, Yu Maw Htwe, Jennifer Toth, Thomas Schaer, Leslie Brewer, Rachel Hilliard, William E. Higgins

**Affiliations:** a Penn State University, School of Electrical Engineering and Computer Science, University Park, Pennsylvania, United States; b Penn State University, College of Medicine, Hershey, Pennsylvania, United States; c University of Pennsylvania, Department of Clinical Studies New Bolton Center, Philadelphia, Pennsylvania, United States

**Keywords:** bronchoscopy, endobronchial ultrasound, image registration, image-guided bronchoscopy, robotics-assisted bronchoscopy, image-guided surgery systems, working channel instruments, multimodal imaging, lung cancer

## Abstract

**Purpose:**

Lung cancer remains the leading cause of cancer death. This has brought about a critical need for managing peripheral regions of interest (ROIs) in the lungs, be it for cancer diagnosis, staging, or treatment. The state-of-the-art approach for assessing peripheral ROIs involves bronchoscopy. To perform the procedure, the physician first navigates the bronchoscope to a preplanned airway, aided by an assisted bronchoscopy system. They then confirm an ROI’s specific location and perform the requisite clinical task. Many ROIs, however, are extraluminal and invisible to the bronchoscope’s field of view. For such ROIs, current practice dictates using a supplemental imaging method, such as fluoroscopy, cone-beam computed tomography (CT), or radial endobronchial ultrasound (R-EBUS), to gather additional ROI location information. Unfortunately, fluoroscopy and cone-beam CT require substantial radiation and lengthen procedure time. As an alternative, R-EBUS is a safer real-time option involving no radiation. Regrettably, existing assisted bronchoscopy systems offer no guidance for R-EBUS confirmation, forcing the physician to resort to an unguided guess-and-check approach for R-EBUS probe placement—an approach that can produce R-EBUS placement errors exceeding 30 deg, an error that can result in missing many ROIs. Thus, because of physician skill variations, biopsy success rates using R-EBUS for ROI confirmation have varied greatly from 31% to 80%. This situation obliges the physician to turn to a radiation-based modality to gather sufficient information for ROI confirmation. We propose a two-phase registration method that provides guidance for R-EBUS probe placement.

**Approach:**

After the physician navigates the bronchoscope to the airway near a target ROI, the two-phase registration method begins by registering a virtual bronchoscope to the real bronchoscope. A virtual 3D R-EBUS probe model is then registered to the real R-EBUS probe shape depicted in the bronchoscopic video using an iterative region-based alignment method drawing on a level-set-based optimization. This synchronizes the guidance system to the target ROI site. The physician can now perform the R-EBUS scan to confirm the ROI.

**Results:**

We validated the method’s efficacy for localizing extraluminal ROIs with a series of three studies. First, for a controlled phantom study, we observed that the mean accumulated position and direction errors (accounting for both registration phases) were 1.94 mm and 3.74 deg (equivalent to 1.30 mm position error for a 20 mm biopsy needle), respectively. Next, for a live animal study, these errors were 2.81 mm and 4.79 deg (2.41 mm biopsy needle error), respectively. For 100% of the ROIs considered in these two studies, the method enabled visualization of an ROI via R-EBUS in under 3 min per ROI. Finally, initial operating-room tests on lung cancer patients indicated the method’s efficacy, functionality, efficiency, and safety under standard clinical conditions.

**Conclusions:**

The method offers a quick, low-cost, radiation-free approach for examining peripheral extraluminal ROIs using R-EBUS. Although our studies focused on R-EBUS as the supplemental working channel instrument, the proposed method has general applicability to any clinical bronchoscopic task requiring a working channel instrument. Thus, the method has the potential to improve the efficiency and efficacy of bronchoscopic procedures for lung cancer patients.

## 1 Introduction

Lung cancer remains the leading cause of cancer death.^1^ This has driven a critical need for methods that help manage peripheral regions of interest (ROIs) in the lungs, be it for cancer diagnosis, staging, or treatment.^2^

The state-of-the-art approach for managing peripheral ROIs entails bronchoscopy, aided by a computer-based assisted bronchoscopy system. Two types of assisted bronchoscopy systems exist:^3^^–^^5^ (1) the now common image-guided bronchoscopy systems, whereby the physician maneuvers the bronchoscope based on system guidance, and (2) the emerging robotics-assisted bronchoscopy systems, whereby a robot maneuvers the bronchoscope under a physician control.

To perform a bronchoscopy, the physician first identifies a clinical site of interest on a patient’s three-dimensional (3D) chest computed tomography (CT) imaging scan. A procedure plan is then derived from the CT scan consisting of an airway guidance route leading to an ROI representing the site.^3^^,^^6^ Next, during the live bronchoscopic procedure, the physician performs a two-step process:

1. Navigate the bronchoscope through the airway tree to the preplanned final airway nearest the target ROI, with navigational guidance provided by the assisted bronchoscopy system.
2. Confirm the ROI’s location and perform the requisite clinical task.

Fundamentally, to provide navigational guidance, an assisted bronchoscopy system exploits the concept of virtual bronchoscopy (VB), whereby the live procedural bronchoscopic video is correlated to CT-based VB views derived from a “virtual” bronchoscope that mimics the video images produced by the “real” bronchoscope.^7^^–^^10^ To accomplish synchronization of the real and virtual bronchoscopes, registration is performed between the bronchoscopic video and VB views during guided navigation.^9^^–^^11^ In this way, assisted bronchoscopy systems have proven to boost physician performance for bronchoscope navigation,^4^^,^^5^^,^^12^ thereby alleviating the long-known physician skill variations in navigating a bronchoscope through the airway tree.^13^^,^^14^

Note, however, that bronchoscope navigation is insufficient for localizing the large majority of ROIs that are extraluminal, arising outside the airways. This is because the bronchoscope only images the airway’s endoluminal interior and cannot give definitive information on such an ROI’s precise location. For this reason, the second confirmation step is critical for establishing the ROI location. In current state-of-the-art practice, ROI confirmation is carried out using a supplemental imaging method, such as fluoroscopy, cone-beam CT, or radial endobronchial ultrasound (R-EBUS).^2^^–^^4^^,^^6^^,^^15^^–^^19^ Unfortunately, fluoroscopy and cone-beam CT expose the patient to substantial radiation, can lengthen procedure time, are more susceptible to blur and motion artifacts than standard chest CT, and require a significant equipment expenditure.^20^^,^^21^

R-EBUS, on the other hand, is a safer, quicker, cheaper real-time option for ROI confirmation involving no radiation.^22^^,^^23^ R-EBUS uses a thin, flexible probe with a rotating ultrasound transducer to produce two-dimensional (2D) 360-degree radial images in real time.^5^^,^^24^ To confirm an ROI using bronchoscopy and R-EBUS, the physician first navigates the bronchoscope to the preplanned airway. They then insert the R-EBUS probe into the bronchoscope’s working channel and sweep the probe along the airway wall bordering an extraluminal region to confirm the ROI location.

Unfortunately, existing assisted bronchoscopy systems offer no guidance for R-EBUS confirmation. This forces the physician to resort to an unguided blind guess-and-check R-EBUS scanning process.^25^^,^^26^ An additional source of positional uncertainty arises from inserting the R-EBUS probe into the bronchoscope’s working channel. Contrary to the common assumption that the probe’s position is restricted by the bronchoscope’s narrow distal tip construction, the inserted R-EBUS probe’s actual orientation can vary over 30 deg. Thus, a 10-mm probe insertion depth can result in R-EBUS pose errors exceeding ±5  mm—this is more than enough to entirely miss a typical peripheral lesion having a long axis = 10 mm. (The Appendix gives a brief experiment illustrating this point.) Thus, reported biopsy success rates using R-EBUS for ROI confirmation have varied considerably from 31% to 80%.^27^^–^^29^ Yet, because R-EBUS offers real-time ROI visualization, users of robotics-based bronchoscopy systems often use cone-beam CT to help position the R-EBUS probe for final confirmation, thereby incurring the burdensome drawbacks of cone-beam CT.^16^^,^^17^^,^^19^ Clearly, a critical need exists for a more effective means for deploying R-EBUS.

We propose a two-phase registration method that provides guidance for R-EBUS probe placement. In particular, the method offers image-based guidance to help position and align the R-EBUS probe at an optimal location for examining a target ROI.

To apply the method, we have integrated it into a complete image-guided bronchoscopy system under development by our group.^30^ The physician begins with standard navigational guidance to maneuver the bronchoscope through the airways along the preplanned airway guidance route. When the physician reaches the preplanned location for invoking the R-EBUS probe—i.e., the physician has reached the expected vicinity of the target extraluminal ROI, the guidance system displays a virtual R-EBUS probe in the system’s VB viewer. It also displays a CT-based virtual R-EBUS image depicting the expected R-EBUS view at this site^31^ and alerts the physician to insert the R-EBUS probe into the bronchoscope’s working channel. The physician then inserts the probe until it appears in the bronchoscopic video.

Two-phase registration next occurs. First, using the bronchoscopic video and VB views, the bronchoscope and virtual bronchoscope are registered. Any existing method can be used for this step, e.g., the method of Merritt et al.^11^ This synchronizes the pose of the guidance system’s virtual bronchoscope to the bronchoscope’s current pose. Next, given the registered bronchoscope pose as an initialization, the real and virtual R-EBUS probes are registered. In particular, a virtual 3D R-EBUS probe model is registered to the real R-EBUS probe’s shape as depicted in the bronchoscopic video. To accomplish registration, an iterative region-based alignment of the virtual probe model to the R-EBUS probe’s video shape is done using a level-set-based optimization method. The poses of the guidance system’s virtual bronchoscope and probe model are now synchronized to the real bronchoscope and R-EBUS probe at the planned pose. The physician now performs the R-EBUS scan to visualize and confirm the ROI. In this way, the method offers a quick low-cost radiation-free approach for live extraluminal ROI confirmation.

Although our development and experimental studies focus on R-EBUS, our method could easily be adapted to applications drawing on a different working channel instrument. Many recent applications of bronchoscopy tailored toward lung cancer treatment, assessment, and early detection also draw on a supplemental working channel instrument.^3^^,^^23^^,^^32^^,^^33^ As with R-EBUS, the efficacy of such applications is also limited by: (1) the physician’s skill in using the supplemental instrument and (2) the lack of coordinated guidance for both the bronchoscope and the supplemental instrument.

Section 2 first details the two-phase registration method. Section 3 next gives validation tests and illustrates the method within the context of our image-guided bronchoscopy system. Finally, Sec. 4 gives a discussion and concluding remarks.

## 2 Methods

Our goal is to enable effective examination of peripheral extraluminal ROIs using bronchoscopy and R-EBUS. To help with this goal, we have devised a two-phase registration method for assisting with R-EBUS ROI confirmation and integrated the method into an image-guided bronchoscopy system. Section 2.1 overviews the basic imaging inputs and a top-level summary of system operation. This then provides the context for describing the two-phase registration method in Secs. 2.2–2.4.

### 2.1 Input and System Overview

**Fig. 1:**
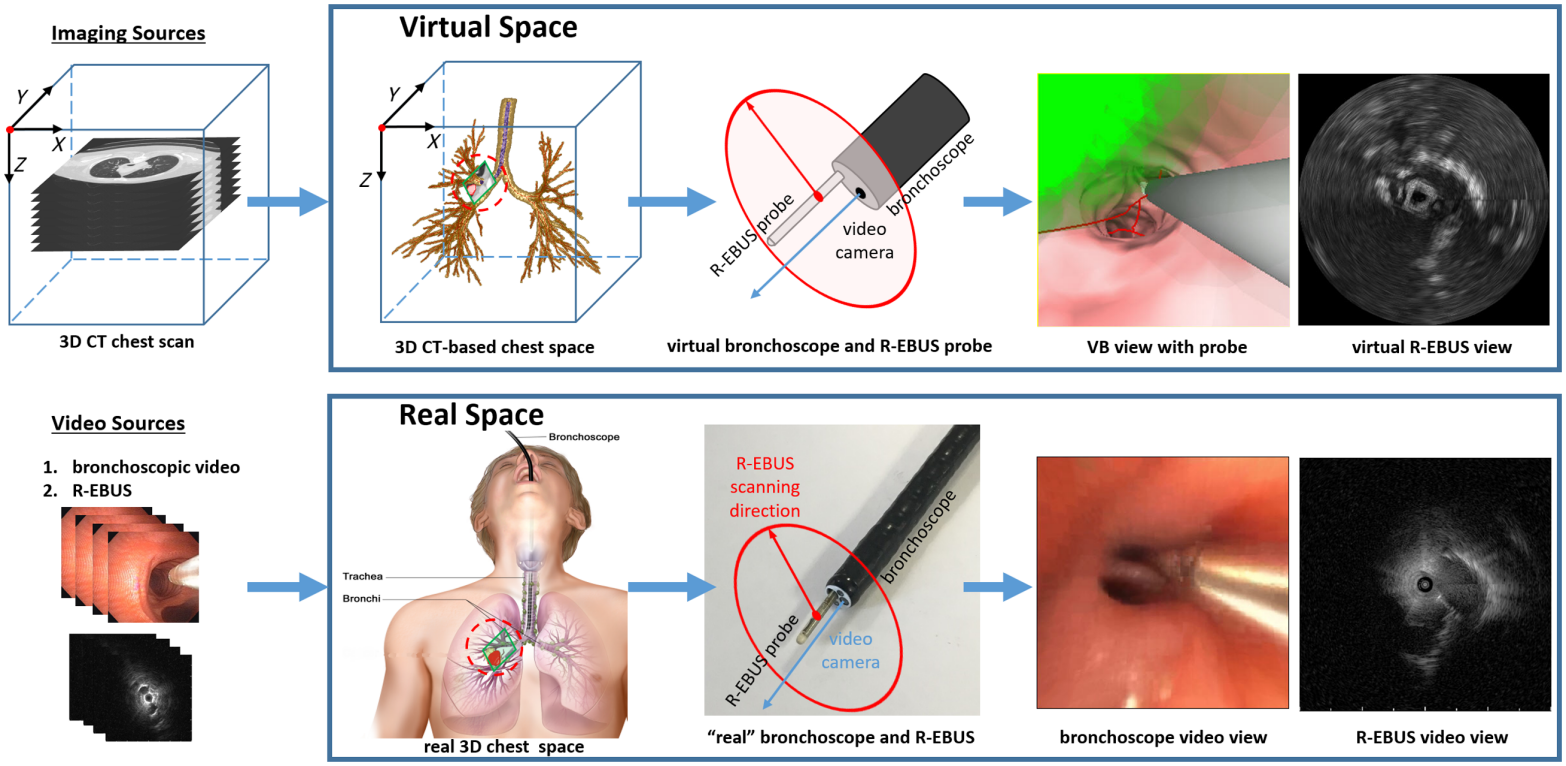
Virtual and real chest spaces illustrating the involved devices and image sources. Regarding the virtual and real space figure blocks, the first column on the far left depicts the manifestations of the 3D chest space. The second column depicts the forms of the bronchoscope/probe device pair. The latter pair of images on the far right illustrate example bronchoscope and R-EBUS images. These image pairs were derived after: (1) navigating toward a left upper lobe lesion; (2) completing the two-phase registration process; and (3) performing the real R-EBUS scan at the final registered pose. The bronchoscope video view depicts the situation after the physician has scanned the desired ROI, with the real R-EBUS probe clearly visible. The corresponding registered VB view depicts the CT-based ROI (green region), predefined by the physician during procedure planning, and the virtual 3D R-EBUS probe model (gray region) registered to the real R-EBUS probe. Both the virtual and real R-EBUS views clearly show the ROI (appears center right). (Patient case 21405.169).

A patient’s high-resolution 3D CT chest scan serves as the main radiologic imaging input to our system. During live bronchoscopy, the system also taps the live picture-in-picture video stream of the bronchoscopy hardware’s main display, depicting both the bronchoscope and R-EBUS probe, as an additional input (Fig. 1). For our tests, the physicians employed standard Olympus bronchoscopy equipment: (a) bronchoscopes having at least a 1.7-mm working channels (models BF-P180 or BF-1TH190) and (b) a 1.4 mm diameter Olympus UM-S20-17S R-EBUS probe.

As is standard for assisted bronchoscopy systems, the clinical work flow for using the system entails (1) off-line procedure planning followed by (2) live guided bronchoscopy in the operating room.^34^^,^^35^ For procedure planning, a set of automatic 3D image analysis operations draw on the patient’s chest CT scan to generate a chest model. The model consists of the airway tree and centerlines, airway endoluminal surfaces, major vasculature (aorta, pulmonary artery), and thoracic cavity. Next, the physician defines an ROI on the CT scan representing the target clinical site of interest. An airway guidance route leading to the ROI is then automatically computed. Well-established previously validated methods are used for all of these operations.^12^^,^^36^^–^^42^ An important feature of the airway guidance route is that guidance cues indicating where and when to invoke the R-EBUS probe for confirming the lesion are also associated with the route.^42^

Given the procedure plan, live bronchoscopy now takes place in the operating room. To perform image-guided bronchoscopy, the system draws on the notion of VB, as generally done by other assisted bronchoscopy systems.^7^^,^^9^^–^^11^ In particular, we consider the physical chest space in two distinct spaces, as shown in Fig. 1:^11^^,^^43^

• A virtual space, established by the 3D CT chest scan, serves as the reference space and provides the guidance information used during the live procedure.
• A real space, derived from the live bronchoscopic and R-EBUS video sources, supplies live intraluminal and extraluminal imaging information.

**Fig. 2:**
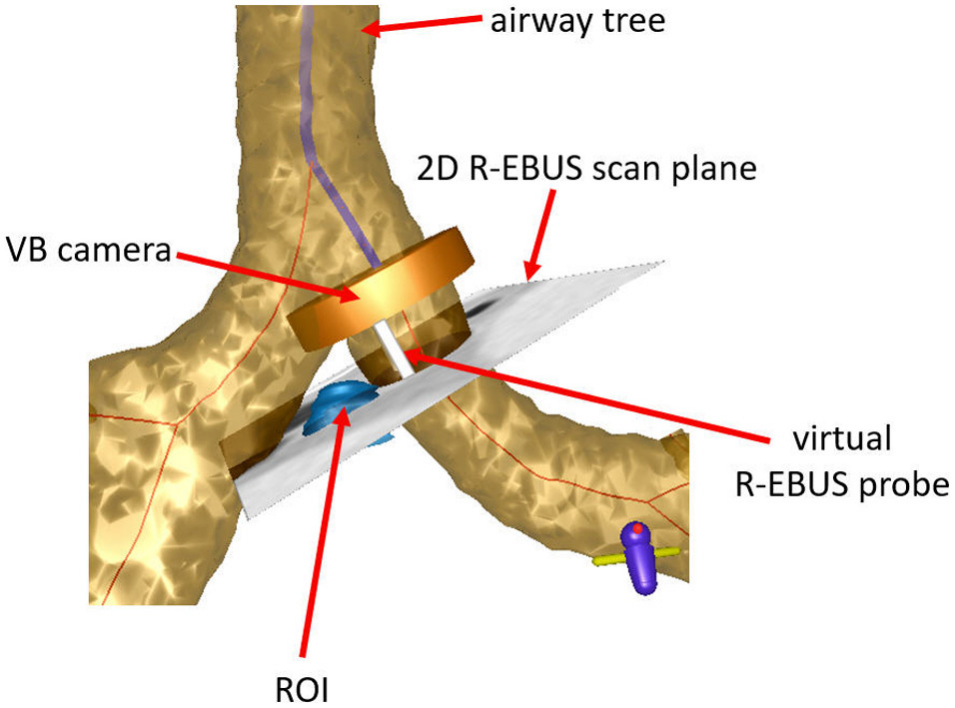
Multimodal virtual device pair featuring models for both the bronchoscope and R-EBUS probe. The figure illustrates the device pair at a location within the 3D CT-based virtual airway tree. The blue line indicates the airway guidance route, whereas the blue region denotes an ROI. The VB camera produces VB views. The virtual R-EBUS probe produces 2D virtual R-EBUS images along the scan plane perpendicular to the device, similar to a real R-EBUS probe.

As a unique element required for our problem, manifestations of both devices—namely, the bronchoscope and R-EBUS probe—are also defined in the virtual space. This results in a virtual multimodal device pair featuring both a virtual bronchoscope and a virtual R-EBUS probe. Figure 1 depicts a schematic view of this device pair, whereas Fig. 2 illustrates the geometry of the two devices in virtual space.

**Fig. 3:**
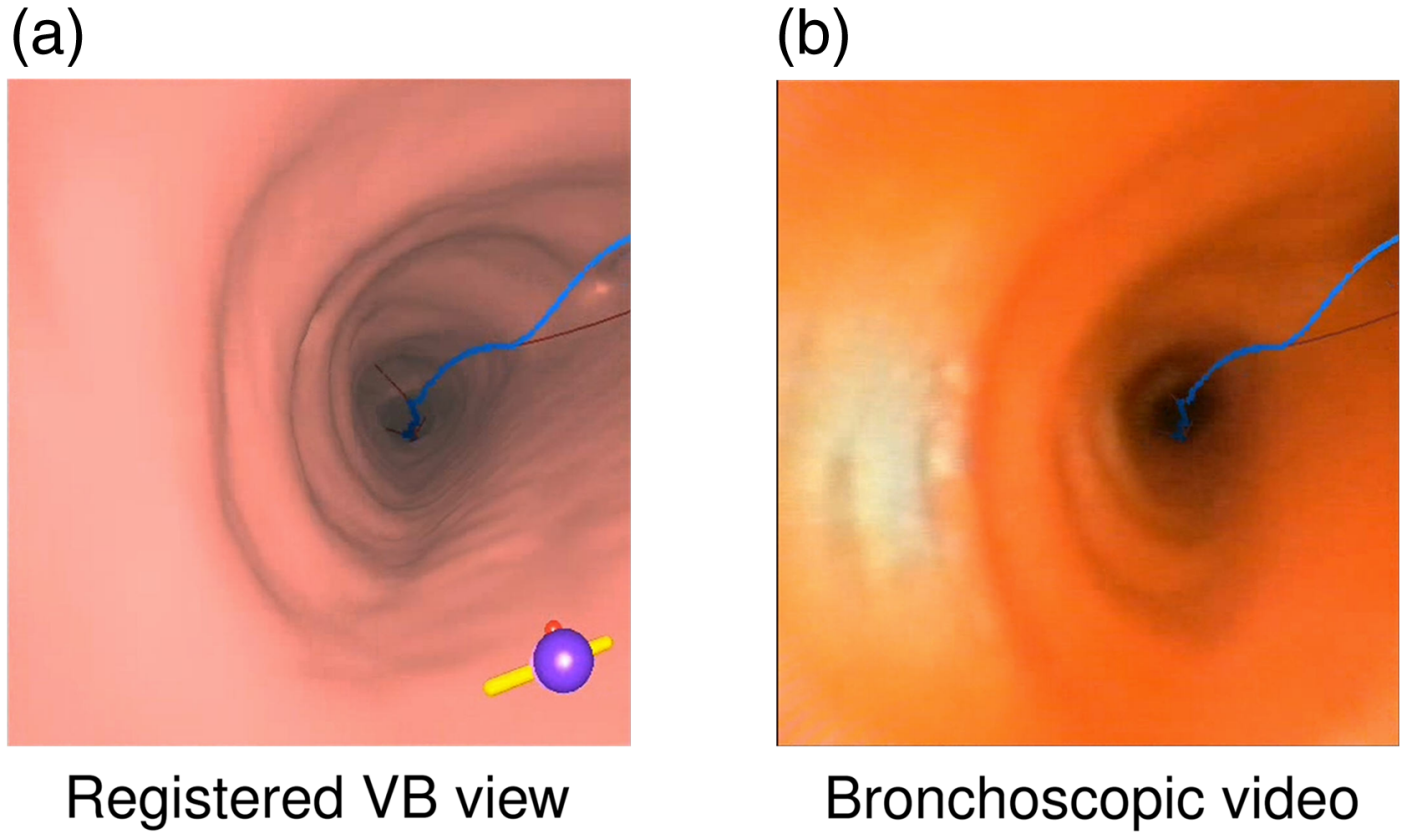
Example registration of the virtual bronchoscope and real bronchoscope during navigation using the method of Merritt et al. (case 21405-3a):^11^ (a) VB view; (b) bronchoscopic video. The blue line indicates the airway guidance route, which is also fused onto the video frame.

To examine an ROI using the system, the physician navigates the bronchoscope through real airways in synchrony with the virtual bronchoscope along the preplanned airway guidance route. Periodic registrations of the virtual and real bronchoscopes are done to synchronize their respective poses. Figure 3 gives a registration example.

Upon reaching the preplanned airway, the physician now performs R-EBUS confirmation, which entails the following:

1. The system displays two images:
  • A VB view that now also depicts the virtual R-EBUS probe. This simulates the insertion of the R-EBUS probe into the bronchoscope’s working channel.
  • A CT-based virtual R-EBUS image that tells the physician what the physician could see if they perform a real R-EBUS scan at the virtual probe’s location.

The guidance system also instructs the physician to insert the R-EBUS probe.

2. The physician inserts the R-EBUS probe into the bronchoscope’s working channel until the probe appears in the bronchoscopic video image.
3. Two-phase registration now occurs to register both virtual devices to the corresponding real devices. The physician can now perform the requisite R-EBUS scan to confirm the ROI. Figure 1 gives examples of the virtual and real images produced after ROI confirmation.

All virtual and real bronchoscope registrations required for navigation and R-EBUS confirmation are done using the Merritt et al.^11^ method (Fig. 3). To enable bronchoscope registration, we calculate the bronchoscope’s calibration parameters in advance so that the virtual bronchoscope’s rendering engine has the same field of view as the real bronchoscope.^44^ Other registration methods used in assisted bronchoscopy systems are certainly viable, be they for VB navigation or electromagnetic navigational bronchoscopy (ENB) systems.^9^^,^^10^ In addition, emerging shape-sensing technology used by robotics-assisted bronchoscopy systems is applicable.^3^^,^^5^

Regarding R-EBUS confirmation, we note in passing that the virtual R-EBUS image is produced using the method of Zhao et al.^31^ Although the virtual image offers evidence that the R-EBUS probe could derive a clear lesion view at this location, it is not used during two-phase registration. Hence, no further discussion is given for this display component. References 31 and 42 gave a complete system description.

The vital operation demanding attention is the two-phase registration method, as now discussed in Secs. 2.2–2.4. Section 2.2 first lays out the 3D geometry as it relates to the chest, bronchoscope tip, and R-EBUS probe. Section 2.3 then discusses the two-phase registration method. Last, Sec. 2.4 highlights implementation details.

### 2.2 Bronchoscope and R-EBUS Probe Geometry

For the methods of this paper, we use a left, posterior, superior 3D coordinate system, where the positive Z-axis extends downward, the positive X-axis extends to the right, and the positive Y-axis extends inward. The online supplement illustrates this coordinate system. Per the standard camera imaging geometry,^45^ we define a pose in this space as a six-dimensional (6D) vector Θ, consisting of a location X pointing in view direction d^11^

Θ=(X,d)=(x,y,z,θ,ϕ,ψ), (1)

where

X=(x,y,z),d=(θ,ϕ,ψ). (2)

In Eq. (2), x, y, and z refer to the respective coordinate values along the X, Y, and Z axes, whereas θ, ϕ, and ψ are the respective Euler angles representing rotations about the X, Y, and Z axes. We assume rotation begins about the Z-axis followed by the Y-axis and X-axis rotations. Next, we define an initial reference pose

$$\Theta_{0}=(X_{0},d_{0})=(0,0,0,0,0,0)$$

situated at the coordinate system’s origin, where $X_{0}=(0,0,0)$ is the origin and $d_{0}=(0,0,0)$ is the canonical view direction.

For the discussion to follow, we specify the locations and view directions of the bronchoscope and R-EBUS probe by distinct poses of the form in Eq. (1). To consider the movements of a device from one pose to another, we apply the standard rigid body transformation T given by the 4×4 homogeneous matrix

$$\begin{array}{c} T \end{array}=[\begin{array}{cc} R & t\\ 0^{T} & 1 \end{array}]=[\begin{array}{cccc} r_{11} & r_{12} & r_{13} & t_{x}\\ r_{21} & r_{22} & r_{23} & t_{y}\\ r_{31} & r_{32} & r_{33} & t_{z}\\ 0 & 0 & 0 & 1 \end{array}],$$ (3)

where 3×3 sub-matrix R represents the rotation, 3×1 column matrix t represents the translation, $0^{T}$ is a 1×3 vector of zeros, and $[\cdot {]}^{T}$ denotes matrix transpose. To apply transformation Eq. (3) to a device at pose Θ with location X, we represent the location in homogeneous coordinates

$$\overset{˜}{X}=[\begin{array}{c} X\\ 1 \end{array}]=[\begin{array}{c} x\\ y\\ z\\ 1 \end{array}],$$

where “∼” quantities represent homogeneous coordinate analogs throughout. Thus, the transformation of a device at reference pose $\Theta_{0}$ to a new pose Θ can be expressed as

$${\overset{˜}{X}}_{\Theta }=T_{\Theta }{\overset{˜}{X}}_{\Theta_{0}}=[\begin{array}{cc} R_{\Theta } & t_{\Theta }\\ 0^{T} & 1 \end{array}]\cdot [\begin{array}{c} X_{\Theta_{0}}\\ 1 \end{array}]\;=[\begin{array}{c} R_{\Theta }X_{\Theta_{0}}+t_{\Theta }\\ 1 \end{array}],$$ (4)

where $T_{\Theta }$ represents the transformation from pose $\Theta_{0}$ to Θ, whereas $X_{\Theta_{0}}=X_{0}$ and $X_{\Theta }$ represent the locations of the device at the initial and new poses, respectively.

We can generalize Eq. (3) to define incremental movements of a device along a trajectory (airway guidance route) in 3D space from instances t to t+Δt using

$$\begin{array}{c} T(t) \end{array}=[\begin{array}{cc} R(t) & t(t)\\ 0^{T} & 1 \end{array}].$$ (5)

Alternatively, we will use the concept of twists to represent such incremental rigid body movements along a trajectory, with the twist set derived from 4×4 matrix^46^^,^^47^

ξ^(t)=[w^(t)v(t)0T0]=[0−ω3ω2v1ω30−ω1v2−ω2ω10v30000], (6)

where w^ is a 3×3 skew-symmetric matrix and v is a 3×1 vector derived from Eq. (5) via

w^(t)=dR(t)dt·RT(t) (7)

v(t)=dt(t)dt−w^(t)t(t). (8)

Thus, a twist is given by the 6D twist coordinate vector

$$\xi (t)=[\begin{array}{c} w(t)\\ v(t) \end{array}]=[\omega_{1}\omega_{2}\omega_{3}v_{1}v_{2}v_{3}{]}^{T},$$ (9)

where angular and linear velocities, w(t) and v(t), indicate the rotational and translational parts of the rigid body motion, respectively. Note that ξ^(t) of Eq. (6) acts as a tangent vector along the trajectory at instance t in that it denotes a first-order approximation to the transformation from t to t+Δt

T(t+Δt)≈T(t)+ξ^(t)T(t)Δt=T(t)+ξ^(t)T(t)if  Δt=1.

Finally, by combining Eqs. (4)–(9), it can be shown that $T_{\Theta }$ can be represented using twists as the matrix exponential^46^

$$T_{\Theta }=\exp(\xi ).$$ (10)

We now define the specific transformations needed for our two-device problem. During assisted bronchoscopy, we use the standard assumption that the bronchoscope camera’s pose changes incrementally during guidance along the airway guidance route from some known pose

$$\Theta_{a}=(x_{a},y_{a},z_{a},\theta_{a},\phi_{a},\psi_{a})$$

to a new unknown pose

$$\Theta_{b}=(x_{b},y_{b},z_{b},\theta_{b},\phi_{b},\psi_{b}),$$

$\Theta_{b}$ is readily obtained via virtual-to-real bronchoscope registration, as stated earlier.^11^ Per Eqs. (4) and (5), this gives the relation

$${\overset{˜}{X}}_{\Theta_{b}}=T_{\Theta_{ba}}{\overset{˜}{X}}_{\Theta_{a}}=T_{\Theta_{b}}{\overset{˜}{X}}_{\Theta_{0}},$$ (11)

where $T_{\Theta_{ba}}$ transforms pose $\Theta_{a}$ to $\Theta_{b}$, $T_{\Theta_{b}}$ transforms initial reference pose $\Theta_{0}$ to $\Theta_{b}$, and

$${\overset{˜}{X}}_{\Theta_{a}}=[x_{a}\;y_{a}\;z_{a}\;1{]}^{T}\;\text{and}\;{\overset{˜}{X}}_{\Theta_{b}}=[x_{b}\;y_{b}\;z_{b}\;1{]}^{T}$$

are homogeneous coordinate forms for the 3D bronchoscope camera locations.

**Fig. 4:**
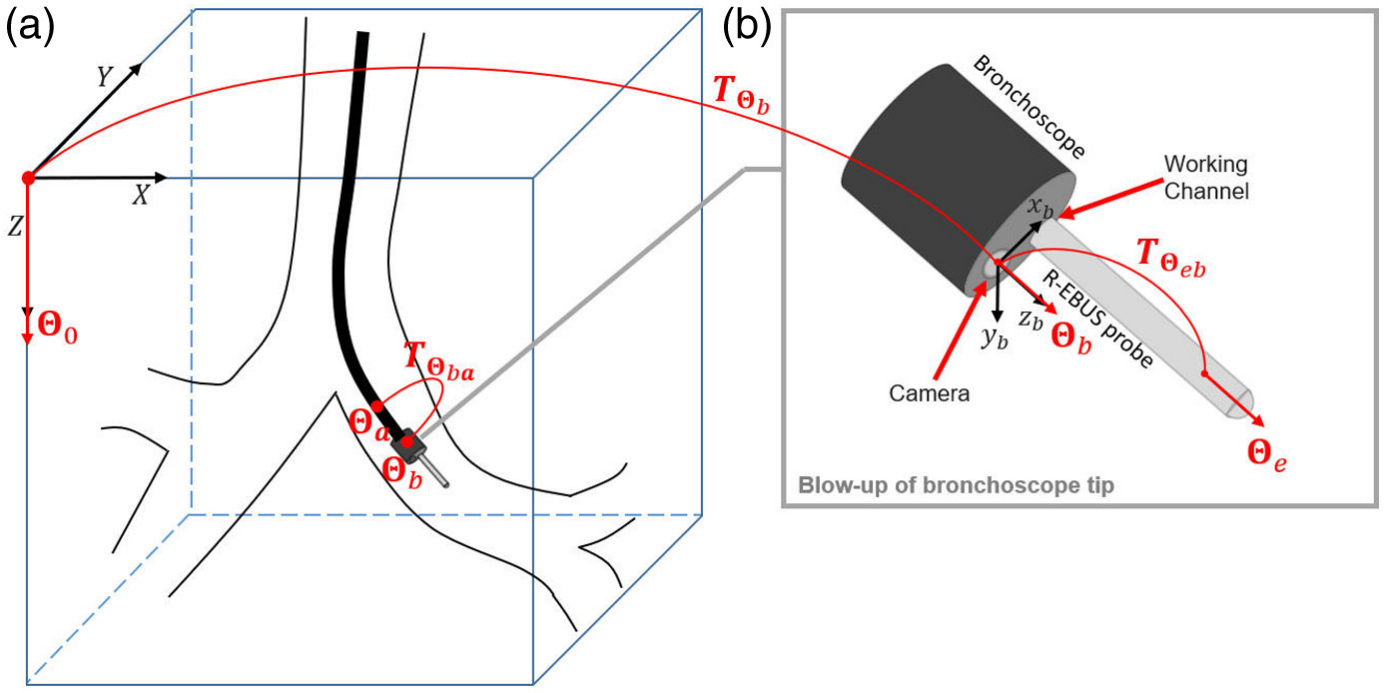
Bronchoscope and R-EBUS probe geometry in 3D chest space after the physician inserts the probe during R-EBUS confirmation. (a) 3D space depicting the airway tree, with the bronchoscope and inserted R-EBUS probe. The last known registered bronchoscope camera pose $\Theta_{a}$ and current unknown bronchoscope camera pose $\Theta_{b}$ are labeled, along with transformation $T_{\Theta_{ba}}$ (from pose $\Theta_{a}$ to pose $\Theta_{b}$). (b) (gray square): blow-up of the local region around the bronchoscope tip centered at $X_{\Theta_{b}}$, with transformation $T_{\Theta_{eb}}$ (from bronchoscope camera pose $\Theta_{b}$ to R-EBUS probe pose $\Theta_{e}$).

**Fig. 5:**
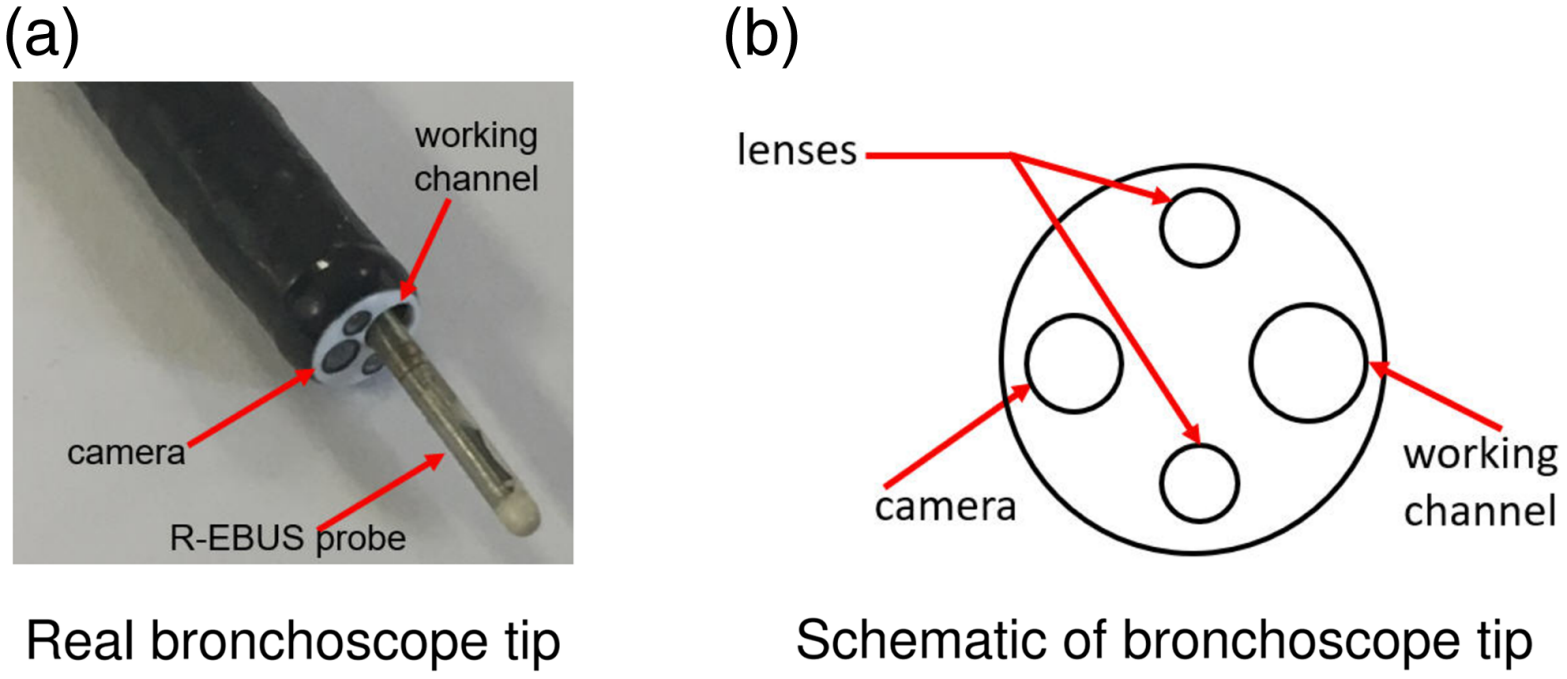
Geometry for the bronchoscope tip components. Panel (a) shows the Olympus BF-P180 bronchoscope (1.7 mm working channel) with the 1.4-mm diameter Olympus UM-S20-17S R-EBUS probe inserted into the working channel. Panel (b) depicts a schematic of the components constituting the device tip.

Now, we consider the situation when the physician has reached the final airway of interest and has inserted the R-EBUS probe into the bronchoscope’s working channel. That is, the physician has just completed step 2 of R-EBUS confirmation, as summarized earlier. At this juncture, we denote without loss of generality the last known registered pose of the real and virtual bronchoscopes as $\Theta_{a}$ and the current unknown poses of the bronchoscope and inserted R-EBUS probe as $\Theta_{b}$ and

$$\Theta_{e}=(x_{e},y_{e},z_{e},\theta_{e},\phi_{e},\psi_{e}),$$

respectively. See Fig. 4. Note that although the bronchoscope camera and working channel are both situated at the distal end of the bronchoscope and have a known constrained geometry (Fig. 5), the 1.4-mm diameter R-EBUS probe has freedom of movement within the larger 1.7-mm diameter working channel. In fact, the physician has much leeway in moving and pivoting the probe within the local air space in front of the bronchoscope tip. Therefore, as Fig. 4 shows, two transformations are required to determine the unknown poses:

1. Transformation $T_{\Theta_{ba}}$, to derive bronchoscope pose $\Theta_{b}$ from the known pose $\Theta_{a}$, per Eq. (11). This brings us to the local camera coordinate system’s origin centered at ($x_{b}$, $y_{b}$, $z_{b}$), as given by the current bronchoscope pose $\Theta_{b}$.
2. Transformation $T_{\Theta_{eb}}$ to derive R-EBUS pose $\Theta_{e}$ from pose $\Theta_{b}$, per
  $${\overset{˜}{X}}_{\Theta_{e}}=T_{\Theta_{eb}}{\overset{˜}{X}}_{\Theta_{b}}=T_{\Theta_{eb}}T_{\Theta_{ba}}{\overset{˜}{X}}_{\Theta_{a}},$$ (12)
  where
  $${\overset{˜}{X}}_{\Theta_{e}}=[x_{e}y_{e}z_{e}1{]}^{T}$$
  is the current 3D location of the R-EBUS probe and $T_{\Theta_{eb}}$ denotes the transformation that maps ${\overset{˜}{X}}_{\Theta_{b}}$ to ${\overset{˜}{X}}_{\Theta_{e}}$.

Two-phase registration—step 3 of R-EBUS confirmation—derives the unknown transformations in Eqs. (11) and (12).

### 2.3 Two-Phase Registration Method

**Fig. 6:**
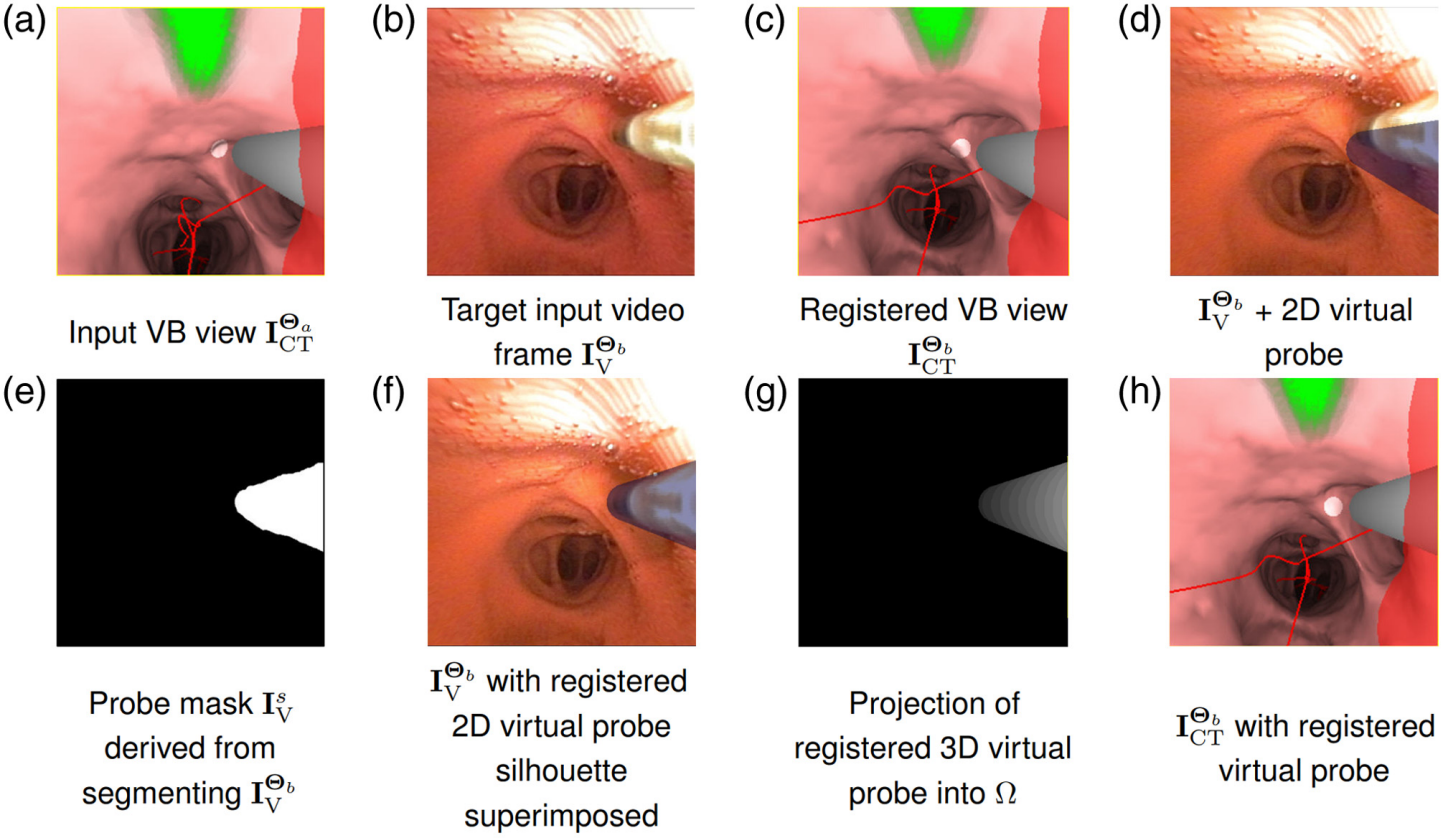
Two-phase registration example. The green, gray, and red regions in the VB views of panels (a), (c), and (h) represent the predefined ROI, the 2D virtual probe model, and nearby major vasculature, respectively. Panels (d) and (f) depict the transparent silhouette of the virtual probe projected onto the real video frame before and after phase 2 of the registration method, respectively. The projection view of panel (g) emphasizes that the projected probe model is a 3D structure, where gray levels indicate the depths of vertices defining the model. Refer to the text in Sec. 2.3 for a full discussion of this figure. (Patient case 20349.3.48). Panel (a) Input VB view $I_{\mathrm{CT}}^{\Theta_{a}}$, Panel (b) Target input video frame $I_{V}^{\Theta_{b}}$, Panel (c) Registered VB view $I_{\mathrm{CT}}^{\Theta_{b}}$, Panel (d) $I_{V}^{\Theta_{b}}+2\text{D}$ virtual probe, Panel (e) Probe mask $I_{V}^{s}$ derived from segmenting $I_{V}^{\Theta_{b}}$, Panel (f) $I_{V}^{\Theta_{b}}$ with registered 2D virtual probe silhouette superimposed, and Panel (g) Projection of registered 3D virtual probe into $\Omega (\text{h})\;I_{\mathrm{CT}}^{\Theta_{b}}$ with registered virtual probe.

To motivate the method, notice that information on the real R-EBUS probe’s pose $\Theta_{e}$ can be gleaned from its image in the bronchoscopic video. Regarding virtual space, we draw on a graphical R-EBUS probe model with a pose that is controlled and known by the guidance system. These points, along with the ability to register the poses of the virtual and real bronchoscopes, drive our two-phase registration method. We refer to Fig. 6 throughout this subsection to illustrate various steps in the method.

The inputs to the method are:

• 2D VB view $I_{\mathrm{CT}}^{\Theta_{a}}$ situated at known pose $\Theta_{a}$ and depicting the virtual R-EBUS probe. $I_{\mathrm{CT}}^{\Theta_{a}}$ reflects the guidance system’s state after the virtual probe has been inserted into the working channel.
• Target bronchoscopic video frame $I_{V}^{\Theta_{b}}$, captured by the system after the physician inserts the R-EBUS probe.

Figures 6(a) and 6(b) give examples of these inputs. For this situation, the real space poses of the bronchoscope $\Theta_{b}$ and R-EBUS probe $\Theta_{e}$ are unknown (see Fig. 4). They are found via the following method:

1. Phase 1—bronchoscope registration: The virtual bronchoscope is aligned to the real bronchoscope by registering the VB view $I_{\mathrm{CT}}^{\Theta_{a}}$ to the target bronchoscopic video frame $I_{V}^{\Theta_{b}}$. This synchronizes the pose of the guidance system to the bronchoscope’s pose $\Theta_{b}$.
2. Phase 2—R-EBUS probe registration: Using the registered bronchoscope pose $\Theta_{b}$ as an initialization, the virtual 3D R-EBUS probe’s graphical model is registered to the real R-EBUS probe’s 2D shape visible in the target video frame $I_{V}^{\Theta_{b}}$. This entails two operations:
  (a) segment the R-EBUS probe’s 2D shape depicted in video frame $I_{V}^{\Theta_{b}}$
  (b) perform virtual-to-real R-EBUS probe registration to ascertain the R-EBUS pose $\Theta_{e}$ relative to the bronchoscope at pose $\Theta_{b}$. For this operation, we adapt a region-based alignment method proposed by Tjaden et al. to our problem.^48^

The 3D positions of the guidance system’s virtual bronchoscope and probe model are now synchronized to the real bronchoscope and R-EBUS probe, i.e., the unknown transformations of Eqs. (11) and (12) are determined. The physician now scans the airway wall to confirm the ROI.

Phase 1 bronchoscope registration employs the standard registration operation used during bronchoscope navigation, with our results drawing on Merritt et al.^11^ After registration, transformation $T_{\Theta_{ba}}$ from Eq. (11) becomes known so that the real and virtual bronchoscopes are now registered at the known pose $\Theta_{b}$. This gives a registered VB view $I_{\mathrm{CT}}^{\Theta_{b}}$, as illustrated in Fig. 6(c). Note that the virtual R-EBUS probe remains rigid and moves in tandem with the virtual bronchoscope during this operation. Figure 6(d) depicts a 2D projection of the 3D virtual probe in Fig. 6(c) onto the target video frame $I_{V}^{\Theta_{b}}$—clearly, the virtual and real probes are not registered.

Phase 2 R-EBUS probe registration now registers the virtual and real R-EBUS probes to give the unknown transformation $T_{\Theta_{eb}}$ per Eq. (12). This involves segmenting the probe in the target video frame $I_{V}^{\Theta_{b}}$ to give a segmented frame $I_{V}^{s}$ and then performing probe registration between $I_{V}^{s}$ and the virtual R-EBUS probe model. We elaborate on these two steps below.

As with the virtual and real bronchoscopes, both the virtual and real probes abide by the same bronchoscope tip configuration, as shown in Figs. 4 and 5. To represent the R-EBUS probe for later registration, we create two region-based forms: (1) a 3D virtual R-EBUS probe model with an initial known 6D pose that is determined by the device pair’s pose as represented by a registered 2D VB view $I_{\mathrm{CT}}^{\Theta_{b}}$; and (2) a 2D segmentation $I_{V}^{s}$ of the real probe’s shape as it appears in target frame $I_{V}^{\Theta_{b}}$.

**Fig. 7:**
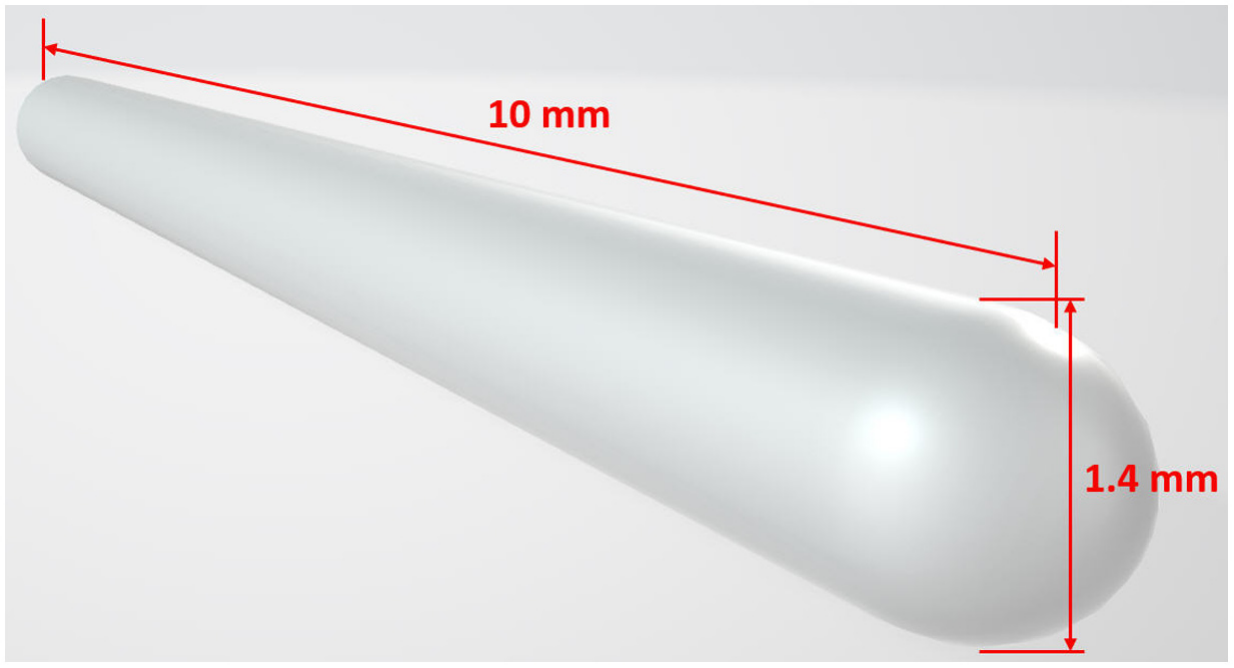
Olympus UM-S20-17S R-EBUS probe mesh model generated from Blender software.^49^ The virtual R-EBUS has a length of 10 mm and a diameter of 1.4 mm.

The virtual R-EBUS probe employs a 3D mesh model generated by the Blender software package, as shown in Fig. 7.^49^ The model is comprised of a dense set of 2382 vertices equally sampled across the visible surface to minimize shape approximation. The model consists of a round-capped cylinder with dimensions that match those of the Olympus UM-S20-17S R-EBUS probe (Fig. 5). In particular, the model has a 1.4-mm diameter, which is the same as the distal end of the Olympus probe. In addition, the virtual probe’s length is constrained to 10 mm, i.e., this is the maximum length that the virtual probe can be “inserted” into the virtual model. Last, a hemispherical cap is added to the model to mimic the shape of the real probe’s distal end. The online supplement illustrates the form of the virtual R-EBUS probe model.

Regarding probe segmentation, note that the registration method of Tjaden et al. combines statistical segmentation and registration simultaneously into a one-step process.^48^ Unfortunately, for our problem, it is difficult to distinguish the partially transparent R-EBUS probe tip from the airway background. This necessitates prior segmentation of the probe before performing registration. Thus, in our implementation, to segment the probe in a bronchoscopic video frame, we draw on Deeplab v3+, a popular deep-learning-based semantic segmentation model,^50^ where our chosen model was pretrained on the ImageNet, MS-COCO, and PASCAL visual object classes (VOC) 2012 datasets,^51^^–^^53^ with an Xception_65 network backbone.^54^ We replaced the network’s last layer to accommodate our two-class video segmentation problem. To derive the model used for our work, we trained, validated, and tested the model with a set of annotated bronchoscopic video frames arising from phantom, animal, and patient studies. The annotations involved two classes: (1) probe (foreground) and (2) airway interior (background). These data depicted the R-EBUS probe over a wide range of poses and insertion lengths. They were collected from multiple bronchoscopes and lung locations.

We imported the pretrained model into our guidance system for live-assisted bronchoscopy. Other methods could certainly be used for this operation.^55^ However, for our relatively constrained problem, Deeplab v3+ gave commendable mean intersection over the union measures of 96.4%, 97.0%, and 93.8% for phantom, animal, and patient test data, respectively. Complete detail appears in Ref. 42. Figure 6(e) depicts an example of automatic segmentation of a given target video frame $I_{V}^{\Theta_{b}}$. Denote this segmented image as

$$I_{V}^{s}(x,y)=\{\begin{array}{ll} 1, & \text{if}\;(x,y)\in \text{segmented probe}\\ 0, & \text{otherwise}, \end{array}$$ (13)

where (x,y) is a pixel location in 2D image domain Ω.

Given the 3D virtual probe model and segmented image $I_{V}^{s}$, we now perform probe registration. For our work, we adapt a region-based alignment method proposed by Tjaden et al.^48^ to our problem. Tjaden’s basic method, which was applied to object tracking in video sequences, tracks the 6D pose (1) of a 3D object as it appears in a video sequence of 2D camera images. To find an object’s pose Θ within a given video frame, the method considers how the object’s intrinsic shape projects onto the frame. To do this, the method solves a curve evolution optimization utilizing a level set function that integrates a shape model.^56^

For our adaptation, we use the known bronchoscope calibration parameters to project the 3D virtual probe model into the 2D domain Ω of $I_{V}^{s}$. This gives a 2D probe silhouette mask that separates domain Ω into a foreground region $\Omega_{F}$, representing the probe, and a background region $\Omega_{B}$, representing the airway interior outside the probe. Thus, the boundary between $\Omega_{F}$ and $\Omega_{B}$ forms the closed curve

C={(x,y)|Φ(x,y)=0}, (14)

representing the shape of the projected virtual probe. In Eq. (14), the shape kernel Φ is given by the level set function

$$\Phi (x,y)=\{\begin{array}{ll} \;d(x,y;C), & (x,y)\in \Omega_{B}\\ -d(x,y;C), & (x,y)\in \Omega_{F}, \end{array}$$ (15)

where the distance function

$$d(x,y;C)=\underset{(x_{c},y_{c})\in C}{\min}\|((x,y)-(x_{c},y_{c})\|$$

equals the minimum distance from a location (x,y)∈Ω to a location $(x_{c},y_{c})\in C$.

The goal now is to align the projected 3D virtual probe, represented by shape kernel Φ, to the segmented probe, represented by $I_{V}^{s}$. For this task, a statistical optimization process varies the virtual probe’s pose in 3D space, such that the discrepancy between the foreground $\Omega_{F}$ and background $\Omega_{B}$ region appearance model statistics are maximized.

For the optimization, we use the density function

$$P(\Phi |I_{V}^{s})=\underset{(x,y)\in \Omega }{\prod }(H(\Phi (x,y))P_{F}+(1-H(\Phi (x,y)))P_{B}),$$ (16)

previously applied in other applications, to describe the shape kernel Φ given $I_{V}^{s}$, where H(·) is the smoothed Heaviside step function and $P_{F}$ and $P_{B}$ are foreground and background region membership probability density functions, respectively.^48^^,^^57^^,^^58^ To capture the local spatial variations of the virtual probe contour C during region alignment, we utilize the localized appearance model suggested in Refs. 59 and 60. In particular, the following averages of local region membership posterior densities

$${\overset{¯}{P}}_{F}(x,y)=\frac{1}{\overset{n}{\underset{i=1}{\sum }}B_{i}(x,y)}\overset{n}{\underset{i=1}{\sum }}P_{F_{i}}(x,y)B_{i}(x,y)$$ (17)

$${\overset{¯}{P}}_{B}(x,y)=\frac{1}{\overset{n}{\underset{i=1}{\sum }}B_{i}(x,y)}\overset{n}{\underset{i=1}{\sum }}P_{B_{i}}(x,y)B_{i}(x,y)$$ (18)

are substituted for $P_{F}$ and $P_{B}$, respectively, in Eq. (16). In Eqs. (17) and (18), $P_{F_{i}}$ and $P_{B_{i}}$ are specified by the histogram over the i’th local region in $I_{V}^{s}$ defined by the masking function

$$B_{i}(x,y)=\{\begin{array}{cc} 1, & (x,y)\in \Omega_{i}\\ 0, & (x,y)\notin \Omega_{i} \end{array}.$$ (19)

Similar to Tjaden et al., each region $B_{i}$ is a circular region (radius = 3) centered over a projected vertex $v_{i}\in C$ of the virtual probe model, where a randomly selected set of n vertices is considered during each iteration of the optimization.^48^

Observe that Φ depends only on C, which in turn only depends on the virtual probe’s pose Θ; i.e., Φ(x,y)→Φ(x,y;Θ). Therefore, by incrementally updating the virtual probe’s pose during optimization, we can evolve C. Assume that $\Theta_{c^{\prime }b}$ is the known initial virtual probe pose relative to the VB camera, as exemplified by Figs. 6(c) and 6(d). That is, a point $X_{\Theta_{b}}$ in the local space relative to the registered bronchoscope camera at known pose $\Theta_{b}$ maps to $X_{\Theta_{c^{\prime }}}$ if it is moved to local probe space, per the transformation

$${\overset{˜}{X}}_{\Theta_{c^{\prime }}}=T_{\Theta_{c^{\prime }b}}{\overset{˜}{X}}_{\Theta_{b}}.$$

Thus, unknown R-EBUS probe pose $\Theta_{e}$ is given by

$${\overset{˜}{X}}_{\Theta_{e}}=T_{\Theta_{ec^{\prime }}}T_{\Theta_{c^{\prime }b}}{\overset{˜}{X}}_{\Theta_{b}},$$ (20)

where $T_{\Theta_{ec^{\prime }}}$ is the transformation capturing the pose difference between the virtual and real probes. The optimization incrementally updates pose $\Theta_{c^{\prime }b}$, which implicitly updates $T_{\Theta_{ec^{\prime }}}$, until convergence. This then aligns the virtual and real probes at pose $\Theta_{e}$, as desired in Eq. (12). However, instead of updating $\Theta_{c^{\prime }b}$ directly, we perform updates using twist coordinates in Eq. (9), i.e., $\xi_{ec^{\prime }}$ undergoes updates in $P(\Phi (x,y;\xi_{ec^{\prime }})|I_{V}^{s})$.

For the optimization, the energy function

$$E(\xi_{ec^{\prime }})=\underset{(x,y)\in \Omega }{\sum }F(x,y;\xi_{ec^{\prime }}),$$ (21)

derived from Eq. (16), is employed, where

$$\begin{array}{c} F(x,y;\xi_{ec^{\prime }})=-\log(H(\Phi (x,y;\xi_{ec^{\prime }})){\overset{¯}{P}}_{F}(x,y)+(1-H(\Phi (x,y;\xi_{ec^{\prime }}))){\overset{¯}{P}}_{B}(x,y)). \end{array}$$

Equation (21) is then expressed in a more tractable form for re-weighted nonlinear least-squares estimation as

$$E(\xi_{ec^{\prime }})=\frac{1}{2}\underset{(x,y)\in \Omega }{\sum }\psi (x,y)F^{2}(x,y;\xi_{ec^{\prime }}),$$ (22)

with weights

$$\psi (x,y)=\frac{1}{F(x,y;\xi_{ec^{\prime }})}.$$ (23)

Equation (22) is then solved iteratively with a standard Gaussian–Newton optimization algorithm by updating the weights [Eq. (23)]. During each step, the algorithm updates the corresponding twist as Δξ^ec′, and the current probe pose can be calculated as the composition of the matrix exponential of $\Delta \xi_{ec^{\prime }}$ with the previous pose estimate via

TΘec′←exp(Δξ^ec′)TΘec′.

More details on the optimization of Eq. (22) appear in Refs. 42 and 48.

For the example of Fig. 6, Figs. 6(f)–6(g) show respectively the registered virtual probe superimposed on video frame $I_{V}^{\Theta_{b}}$ and the 3D probe model at the registered pose projected onto the 2D image plane. Last, Fig. 6(h) depicts the final VB view after both devices have been registered.

### 2.4 Implementation Details

The method and system were developed and tested on a Dell Precision 7920 tower (64-bit Windows 10, 64 GB random access memory (RAM), Intel Xeon Gold 6138 20-core 2.0 GHz), including an NVIDIA RTX 2080 Ti graphics processing unit (GPU)/graphics card and a Matrox ClarityUHD frame grabber (with Mil X library). All software was developed using Visual Studio in C++. The software draws on several libraries, including visualization toolkit (VTK), OpenGL, Qt, OpenCV, and asset import library (ASSIMP). We built the DeepLab v3+ model for probe segmentation with TensorFlow 1.10.0 and NVIDIA’s CUDA 10.0 library to enable real-time use of the model on the GPU.

Patient CT scans were produced by either a Siemens Somatom, Siemens Sensation-40, or Canon Aquilion Prime CT scanner. Each scan consisted of a series of 512×512 axial-plane sections, where section thickness = 0.75 mm, section spacing = 0.5 mm, and axial-plane resolution Δx=Δy<1.0  mm. The Olympus BF-P180 and BF-1TH190 bronchoscopes output true-color video frames of sizes 640×480 and 1920×1080, respectively, at a rate of 30  frames/s. Per the known bronchoscope tip parameters for the Olympus bronchoscopes, the virtual probe’s center is at the initial pose $\Theta_{c^{\prime }}=(x_{c^{\prime }},y_{c^{\prime }},z_{c^{\prime }},\theta_{c^{\prime }},\phi_{c^{\prime }},\psi_{c^{\prime }})=(2.0\;\mathrm{mm},0.4\;\mathrm{mm},0\;\mathrm{mm},0,0,0)$ for probe registration in the local coordinate system of the bronchoscope camera (Figs. 4 and 5). For the densities ${\bar{P}}_{F}$ and ${\bar{P}}_{B}$ defined in Eqs. (17)–(19), we randomly picked the number of vertices n at each iteration (n≤100) for the $B_{i}$ regions.^48^ The optimization ran a maximum of 300 iterations and stopped if the energy function’s value did not change after an iteration.

To apply the system in a live guided R-EBUS bronchoscopy procedure, we first interface our guidance computer to the surgical suite’s bronchoscopy tower. In particular, the computer draws on the live video streams of the bronchoscope and R-EBUS probe. We then invoke the system software, load the precomputed procedure plan, and initialize the system’s display with the bronchoscope in the trachea. The physician then follows the two-step process discussed previously to perform the guided procedure. During this procedure, the physician has access to the standard picture-in-picture bronchoscopy monitor, which shows both the live bronchoscopic video and R-EBUS video and to the guidance computer display. Zhao et al.^30^^,^^42^ gave complete system-level details on this process.

## 3 Results

We now present a series of progressively more demanding studies to ascertain the performance, functionality, and practicality of the two-phase registration method. Section 3.1 first gives a quantitative phantom study. These results help measure the performance and potential efficacy of the method for a controlled situation, free of time pressure, and safety concerns. Section 3.2 presents controlled *in vivo* animal studies, which help to establish the method’s basic feasibility and functionality in a controlled live situation. Finally, Sec. 3.3 illustrates system results for live lung cancer patient procedures, thereby demonstrating the method’s feasibility within the context of the real clinical work flow.

### 3.1 Phantom Study

To assess the performance of the two-phase registration method, we first performed a phantom study drawing on two human airway-tree phantoms used in previous work.^11^^,^^14^^,^^61^ Each phantom represents an exact replica of the segmented airway endoluminal surfaces depicted in a patient’s high-resolution 3D chest CT scan (voxel resolutions Δx, Δy, Δz<0.8  mm). To construct a phantom, we converted the airway endoluminal surface data into an STL graphics file [standard triangle language (STL)]. The STL file was then used by Stratasys, Inc., to fabricate a phantom using acrylonitrile butadiene styrene, a common rigid thermoplastic used in rapid prototyping.^14^

Five target ROIs, three in phantom 1 and two in phantom 2, were defined in the associated CT scans. Four ROIs were synthetically inserted into the CT scans in air regions, whereas one ROI for phantom 2 represented a real anatomical site visible in CT. The inserted ROIs were assigned a value of 100 Hounsfield units (HU). The volume and long axis ranges of these ROIs were [$340\;{\mathrm{mm}}^{3}$, $562\;{\mathrm{mm}}^{3}$] and [11.7 mm, 15.4 mm], respectively. Procedure plans were computed for all ROIs using the CT scans.

**Fig. 8:**
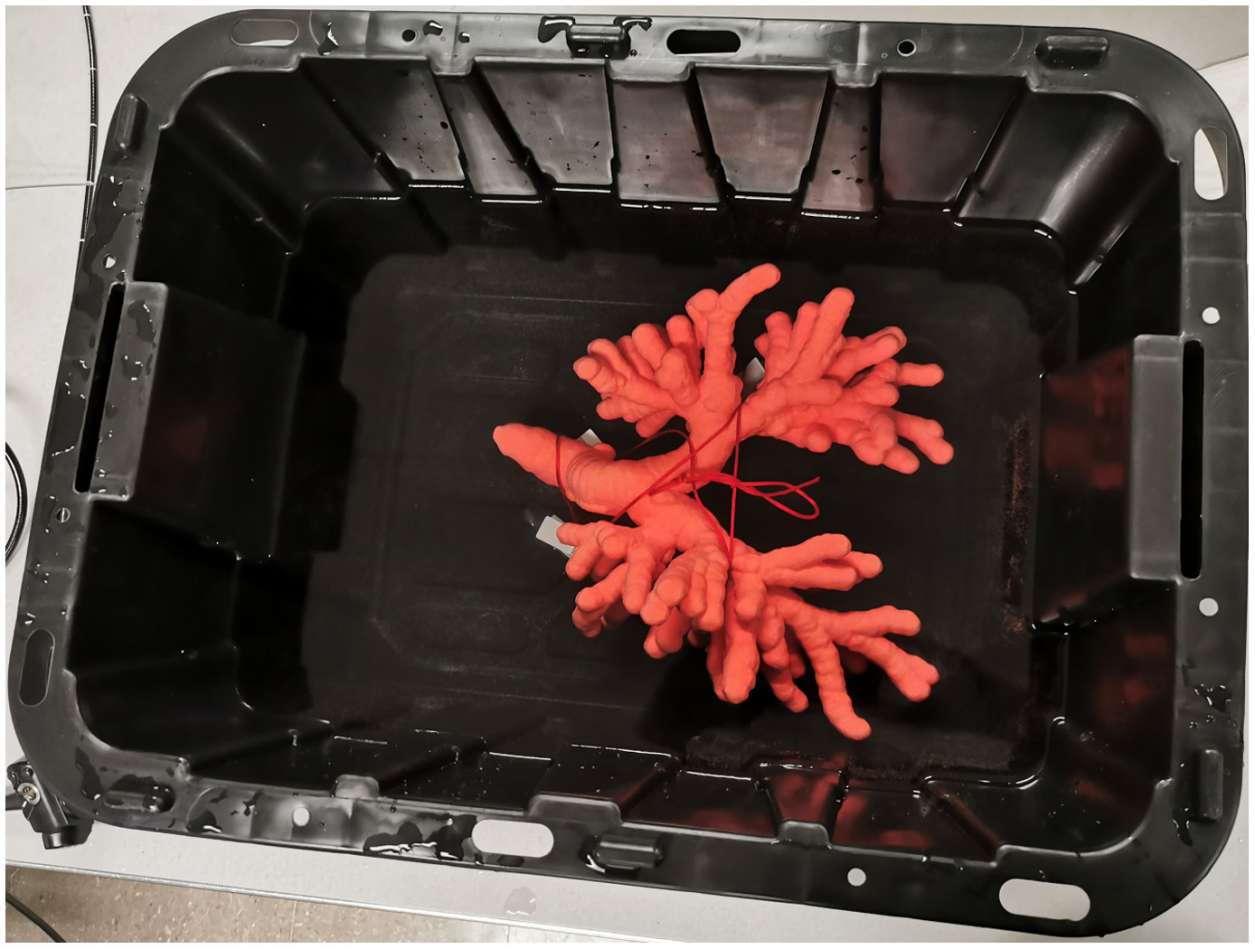
Airway tree phantom 2 secured in a container.

To create a simulated “surgical environment” for performing guided R-EBUS bronchoscopy, we secured a phantom to the bottom of a water-filled container (Fig. 8). We emphasize that the specific goal of the phantom study was to ascertain the accuracy of our two-phase method for registering both the bronchoscope and R-EBUS probe at the final preplanned site of a given ROI. Hence, we did not integrate synthetic ROIs into the phantom set-up, as R-EBUS scanning per se was not a prime goal.

We did, however, still wish to generate R-EBUS images of the phantom during the experiment. Unfortunately, air reflects nearly all ultrasound signals during transmission, making R-EBUS imaging challenging.^62^ To mitigate this issue, we submerged the phantom in water, as done by others previously.^63^ Using this set-up, we could now produce R-EBUS images that clearly depict the airway walls of a phantom. (We note in passing that physicians sometimes use a saline-filled inflatable balloon enclosing the R-EBUS probe to help mitigate the impact of poor coupling.^26^^,^^62^ Our physicians, however, do not take this approach. Instead, they touch the R-EBUS probe against the airway wall adjacent to the extraluminal scanning region of interest to perform a scan.)

Prior to performing the study, we first established ground-truth poses for the bronchoscope and R-EBUS probe at the final preplanned pose for all ROIs. To do this for a given ROI, a technician, in collaboration with two other technicians, first navigated the bronchoscope to the final preplanned airway location. The ground-truth bronchoscope pose $\Theta_{b}^{G}$ was then derived by automatically registering the bronchoscope video view and CT-based VB view via the Merritt et al. method to give an initial $\Theta_{b}$. Although this step typically produces accurate registrations, as an added precaution, they next created an overlay of the registered real and virtual views to help discern any potential mismatch. Based on this display, they made a final manual refinement of the system VB view, if deemed necessary, to arrive at the final match between the VB view and bronchoscopic video. (Quantities such as $\Theta_{b}^{G}$ signify ground truth analogs of $\Theta_{b}$.)

Next, a similar process was undertaken for the final position of the R-EBUS probe. In particular, after the R-EBUS probe was inserted, we segmented the real probe in the video, applied automatic registration between the real probe and the virtual probe model, and then interactively adjusted the virtual probe model’s pose until it best matched the segmented real probe. This established the ground-truth probe pose $\Theta_{e}^{G}$ per Eq. (12) and also a relative pose difference $\Theta_{eb}^{G}$ between the bronchoscope and probe. $\Theta_{eb}^{G}$ enables us to separate the errors arising during the two registration phases. For this study, R-EBUS bronchoscopy was performed using an Olympus BF-P180 bronchoscope and UM-S20-17S R-EBUS probe.

Given the ground truth, we then performed a series of image-guided bronchoscopy trials for each of the five target ROIs designated in the two phantoms. For the study, a trial entailed performing guided R-EBUS bronchoscopy as highlighted in Sec. 2.4, which involves bronchoscope navigation followed by R-EBUS ROI confirmation. Eight trials were completed for each ROI, with all system display video recorded for later analysis.

We then measured the poses $\left(\Theta_{b},\Theta_{eb},\Theta_{e}\right)$ automatically produced by the method for each of the 40 trials. Continuing, we then computed two previously suggested error metrics for each device:^11^^,^^43^ (1) device position difference

$$\text{bronchoscope}:\;e_{p}^{\Theta_{b}}=\|X_{b}-X_{b}^{G}\|\;R\text{-}\mathrm{EBUS}\;\text{probe}:\;e_{p}^{\Theta_{eb}}=\|X_{eb}-X_{eb}^{G}\|,$$ (24)

which measures the Euclidean distance between a device’s ground-truth location and the location derived during bronchoscopy on a given trial and (2) device direction error

$$\text{bronchoscope}:\;e_{d}^{\Theta_{b}}=\cos^{-1}(d_{b}\cdot d_{b}^{G})\;R\text{-}\mathrm{EBUS}\;\text{probe}:\;e_{d}^{\Theta_{eb}}=\cos^{-1}(d_{eb}\cdot d_{eb}^{G}),$$ (25)

which measures the angular orientation error between a device’s ground-truth direction and the direction derived during bronchoscopy. Last, we computed accumulated position and orientation errors for the probe, given by

$$e_{p}^{\Theta_{e}}=\|X_{e}-X_{e}^{G}\|,\;e_{d}^{\Theta_{e}}=\cos^{-1}(d_{e}\cdot d_{e}^{G}),$$ (26)

which measure the total error from two registration phases. In Eqs. (24)–(26), “‖·‖” denotes Euclidean distance, and

$$\begin{array}{l} \Theta_{b}=(X_{b},d_{b}),\;\Theta_{b}^{G}=(X_{b}^{G},d_{b}^{G})\\ \Theta_{eb}=(X_{eb},d_{eb}),\;\Theta_{eb}^{G}=(X_{eb}^{G},d_{eb}^{G})\\ \Theta_{e}=(X_{e},d_{e}),\;\Theta_{e}^{G}=(X_{e}^{G},d_{e}^{G}) \end{array}$$

correspond respectively to the guided and ground-truth pose definitions of the form in Eq. (1).

Table 1 summarizes the registration errors over all 40 trials. From the table, the accumulated position and direction errors were 1.94±1.11  mm and 3.74  deg±1.49  deg, respectively. These measures indicate consistent, accurate performance, well within the criteria suggested by Merritt et al. for successful probe registration ($e_{p}<5.0\;\mathrm{mm}$ and $e_{d}<10\;\deg$).^11^ Furthermore, the maximum position (4.93 mm) and direction (6.94 deg) errors observed after two phases of registration were also within the ranges for successful registration. Therefore, under this controlled experimental setup, the performance of both registration phases was robust across 40 trials for five ROIs.

**Table 1:** Two-phase registration errors over five ROIs and 40 guided bronchoscopy trials for two human phantom cases. The scope and probe error metrics are given by Eqs. (24) and (25), whereas the accumulated error metrics are given by Eq. (26). For each phantom, sample results over eight trials for one ROI are also presented for reference; other ROIs gave similar results.^42^ Quantities of the form A±B signify the mean A± standard deviation B over eight trials for each ROI or over all 40 trials for the overall results. The bracketed values “[·,·]” denote observed ranges for a given error metric.

Case	ROIs	Scope registration		Probe registration		Accumulated error	
		$e_{p}^{\Theta_{b}}$ (mm)	$e_{d}^{\Theta_{b}}$ (°)	$e_{p}^{\Theta_{eb}}$ (mm)	$e_{d}^{\Theta_{eb}}$ (°)	$e_{p}^{\Theta_{e}}$ (mm)	$e_{d}^{\Theta_{e}}$ (°)
Phantom 1	ROI 1	1.05±0.71	3.20±1.61	0.31±0.14	2.06±1.01	1.51±1.40	3.67±1.71
		[0.36, 2.64]	[1.14, 5.45]	[0.08, 0.45]	[0.38, 3.11]	[0.73, 4.93]	[1.14, 6.94]
Phantom 2	ROI 1	1.72±0.77	2.98±0.83	0.59±0.43	2.50±1.43	2.15±1.07	4.43±1.17
		[0.72, 2.81]	[0.22, 4.39]	[0.09, 1.52]	[0.30, 4.51]	[0.83, 3.50]	[3.03, 6.35]
Overall	5 ROIs	1.45±0.68	3.09±1.20	0.38±0.27	2.24±1.03	1.94±1.11	3.74±1.49
		[0.36, 2.81]	[0.22, 5.60]	[0.08, 1.52]	[0.30, 5.03]	[0.45, 4.93]	[0.22, 6.94]

Regarding these results, we note that the errors for the Merritt et al. method for phase-1 bronchoscope registration were small, despite the partial occlusion of the airway structures caused by the R-EBUS probe in the video frame. Similarly, probe registration performed robustly, despite the changing airway structures appearing in the video frames.

Table 2 summarizes the procedure time over 40 trials during guided bronchoscopy. The “Navigate + Insert” time includes the time to navigate the bronchoscope to the final airway and insert the R-EBUS probe, including all technician interactions. This time, of course, depends on the depth of an ROI within the airway tree, i.e., the time increases as the number of airways to navigate increases. The “R-EBUS Register” time includes the time for two-phase registration and technician interactions. Finally, “total procedure time” adds the times for navigation/insertion and R-EBUS registration. Note that the total procedure time does not include the R-EBUS scan time as this depends on the physician’s discretion.

**Table 2:** Bronchoscopy procedure times for the phantom study over 40 trials. Sample results are given for one ROI for each phantom for reference. The “overall” row signifies aggregate results over all 40 trials.

Case	ROI	Navigate + insert	R-EBUS register	Total procedure
		Time (s)	Time (s)	Time (s)
Phantom 1	ROI 1	80.7±8.4	86.1±9.7	166.7±10.0
		[69.9, 92.2]	[68.1, 95.5]	[150.2, 186.3]
Phantom 2	ROI 1	88.5±10.5	80.3±11.5	168.8±11.6
		[64.0, 125.6]	[66.0, 92.3]	[153.5, 195.8]
Overall	5 ROIs	90.5±33.2	79.9±9.8	170.4±34.6
		[60.4, 216.3]	[66.0, 95.5]	[127.4, 299.0]

Notably, for all trials, when an R-EBUS scan was invoked, the probe immediately captured a clear view of an ROI, a major indicator of registration success. The mean procedure time for an ROI was 170.4±34.6  s, or under 3 min per ROI. This time increases as navigation time increases, with longer times associated with deeper ROIs.

Bronchoscope registrations computed by Merritt et al. occurred faster than the 30  frames/s real-time rate.^11^ Probe segmentation required only 0.05 to 0.20 s per frame. Last, the probe-registration optimization proved to be the slowest operation, requiring 10 to 20 s per frame to run the complete 300 iterations; many situations, however, needed many fewer iterations. Thus, ≥88% of the measured mean procedure time (150 of 170 s) involved human-driven device maneuvers and technician interactions (freezing virtual/real bronchoscope views at successive airway endpoints during navigation, selecting an R-EBUS frame to segment, manually invoking R-EBUS probe segmentation, toggling the bronchoscope display’s picture-in-picture mode during R-EBUS registration). Overall, the procedure time is commensurate with typical assisted bronchoscopy systems, with a major notable gain in R-EBUS confirmation success rate.^61^^,^^64^

**Fig. 9:**
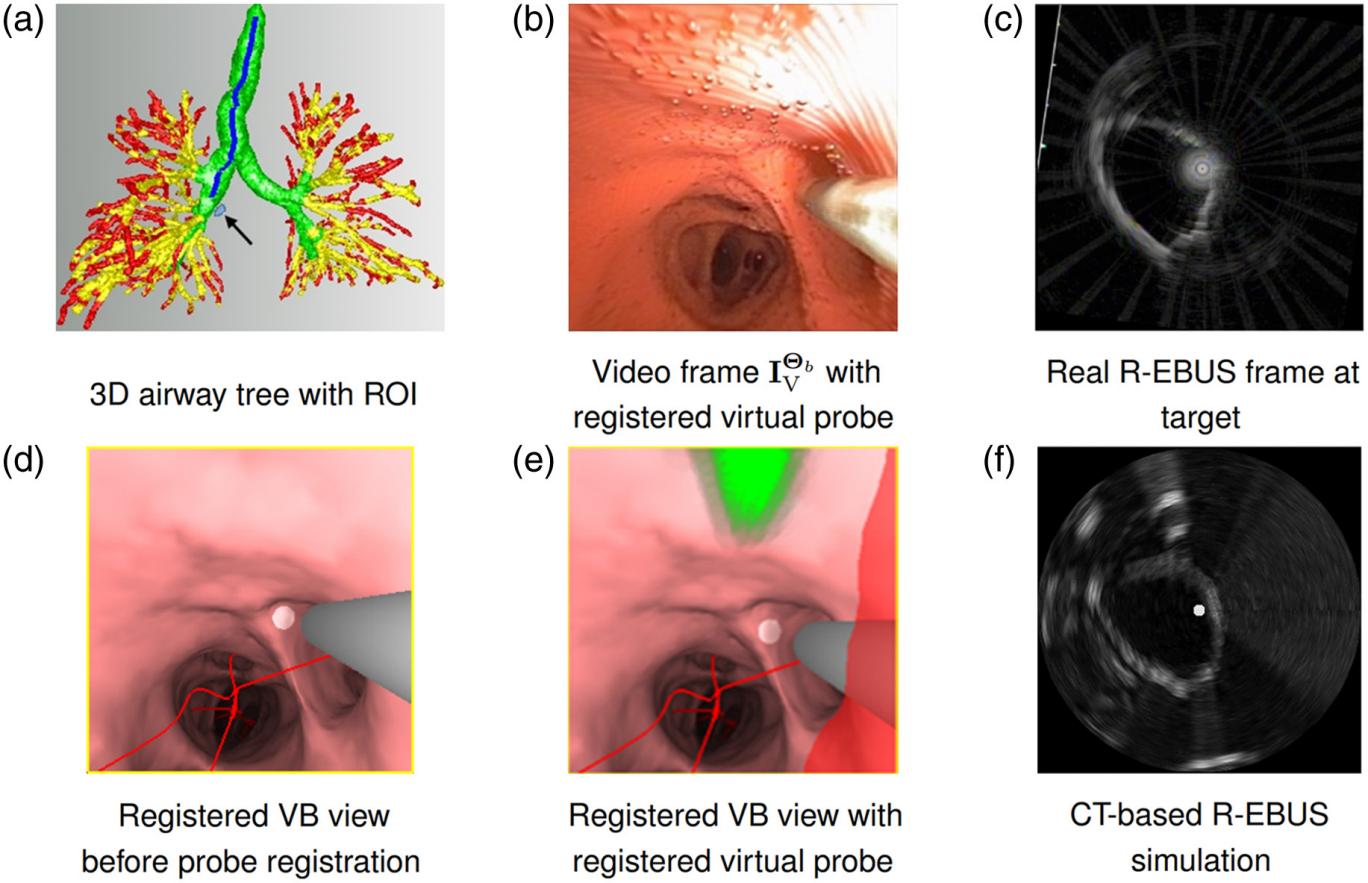
Two-phase RP-EBUS registration example for phantom 2, ROI 4. Panel (a) depicts the CT-based rendered airway tree, with right-lower-lobe ROI 4 rendered in blue (black arrow points to ROI). Panel (b) depicts the real video frame with the R-EBUS probe, whereas panel (c) depicts the corresponding real R-EBUS frame at this scanned location. (Note that the VB view of panel (d) is not used by the system display.) Panel (d) shows the registered VB view before the virtual R-EBUS probe is registered, whereas panel (e) illustrates the virtual devices (bronchoscope and R-EBUS probe) after two-phase registration. Panel (f) depicts the corresponding simulated R-EBUS view corresponding to the device locations shown in panel (e).

Figure 9 gives example results for phantom 2 after completing two-phase registration and performing the R-EBUS scan. Panels (b) and (e) clearly show how the bronchoscope and R-EBUS probe are registered in both the real and virtual spaces. In addition, the similarity between the real and simulated R-EBUS views at this registered position [panels (c) and (f)] corroborates this successful registration. For Fig. 9 and all other figures in Sec. 3, the green, gray, and red regions in the VB views denote a predefined ROI, the 2D virtual probe, and the major vasculature, respectively, as shown in Figs. 1 and 6. In addition, for the rendered 3D airway tree views, the dark blue lines denote the preplanned airway guidance route, whereas the green, yellow, and red regions denote how well a bronchoscope can fit into the indicated airway:^42^ green, fits easily; yellow, tight fit; red, bronchoscope cannot fit.

### 3.2 Animal Studies

We next performed controlled animal studies as a live system test under *in vivo* circumstances. The animal studies were performed under the University of Pennsylvania PennVet IACUC protocol 806312, approval date of August 2020. All animal studies were carried out in accordance with the National Research Council’s Guide for the Care and Use of Laboratory Animals.

Two 40-kg female pigs were considered for the studies. A veterinary surgeon bronchoscopically created synthetic ROIs, visible in CT, by injecting a mixture of carboxymethylcellulose and Omnipaque (GE Healthcare) through a needle inserted into the bronchoscope’s working channel. The surgeon created the ROIs by pushing the needle through airways at various locations in the periphery of an animal’s left and right lung fields. Seven extraluminal ROIs were inserted into the two pigs, 3 for pig #1 and 4 for pig #2, with both pigs having at least one ROI in each lung. For the seven ROIs, the ROI volume and long axis ranges were [$219\;{\mathrm{mm}}^{3}$, $1503\;{\mathrm{mm}}^{3}$] and [11.5 mm, 41.2 mm], respectively. The ROIs were situated near airways at depths ranging from generations 3 to 7 (trachea = generation 1).

A chest CT scan, having resolution comparable to human chest CT scans, was then acquired with a Samsung CereTom portable 8-slice veterinary CT scanner. Each CT scan consisted of 512×512 sections (pig #1, 264 sections; pig #2, 160 sections) with resolution ΔX=ΔY=0.49  mm and ΔZ=1.25  mm. Next, given a CT scan, a procedure plan was generated for each ROI, using the guidance computer software.

Assisted bronchoscopy was then performed, with an animal held under general anesthesia during a study. The physician again employed the Olympus BF-P180 bronchoscope and UM-S20-17S R-EBUS probe for a study. For each ROI, the physician first navigated the bronchoscope to the final target airway and then invoked the R-EBUS probe at the preplanned final location, based on system guidance. All raw bronchoscope display video, which features both the bronchoscopic video stream and R-EBUS video stream in an integrated picture-in-picture display, and the guidance-computer display video were recorded for later analysis.

To generate quantitative results, we first gleaned ground truth results retrospectively. To do this, for a particular ROI, the technicians first replayed the recorded raw bronchoscope video corresponding to the procedure performed for the ROI. When they reached the video frame depicting the best positions for both the bronchoscope and R-EBUS probe for scanning the ROI, they designated this frame as the ground-truth video view for this ROI. Procedures identical to those followed for the phantom study were then performed: (1) register the virtual bronchoscope to the video frame; (2) refine the bronchoscope location, if necessary, using an overlay of the real and virtual bronchoscope views; (3) segment the R-EBUS probe in the video frame; (4) register the virtual R-EBUS probe model to the segmented R-EBUS probe; (5) refine, as necessary. This established ground truth values of $\Theta_{b}^{G}$, $\Theta_{e}^{G}$, and $\Theta_{eb}^{G}$ for all ROIs. We emphasize that the technicians did not consult the recorded computer display video, which contains the actual live registration results, to produce the ground truth data. Given the ground truth data, we then completed the quantitative study.

Table 3 gives error metric results for these studies. The physician was able to scan all ROIs successfully after completing two-phase registration. The accumulated position and direction errors for these live studies were 2.81±1.33  mm and 4.79  deg±1.92  deg, respectively. These errors were 10% to 25% higher than the phantom study errors ($e_{p}^{\Theta_{e}}=1.94\pm 1.11\;\mathrm{mm}$ and $e_{d}^{\Theta_{e}}=3.74\;\deg\pm 1.49\;\deg$), probably due to live breathing and heart motion. Nevertheless, they were again well within the range of the Merritt success criteria.

**Table 3:** Two-phase registration errors for animal studies over seven ROIs. Scope and probe error metrics are given by Eqs. (24) and (25); accumulated error metrics are given in Eq. (26).

Scope registration		Probe registration		Accumulated error	
$e_{p}^{\Theta_{b}}$ (mm)	$e_{p}^{\Theta_{b}}$ (°)	$e_{p}^{\Theta_{eb}}$ (mm)	$e_{d}^{\Theta_{eb}}$ (°)	$e_{p}^{\Theta_{e}}$ (mm)	$e_{d}^{\Theta_{e}}$ (°)
1.74±0.62	3.63±1.40	0.45±0.19	2.46±0.54	2.81±1.33	4.79±1.92
[0.90, 2.41]	[1.50, 5.34]	[0.25, 0.76]	[1.30, 3.30]	[1.51, 4.62]	[1.59, 6.15]

Regarding procedure time, the mean “navigation + insert” and “R-EBUS register” times were 61.7±10.4  s and 68.8±11.0  s, respectively, over seven ROIs. As the pig’s lungs are not quite as large as the human’s, the navigation time overall tended to be less than for the human phantom study. In addition, we noted that the mean R-EBUS scan time per ROI was 32.1±9.9  s. Thus, the mean total procedure time = 162.7±18.3  s, including the ROI scan time, was comparable to the phantom study.

**Fig. 10:**
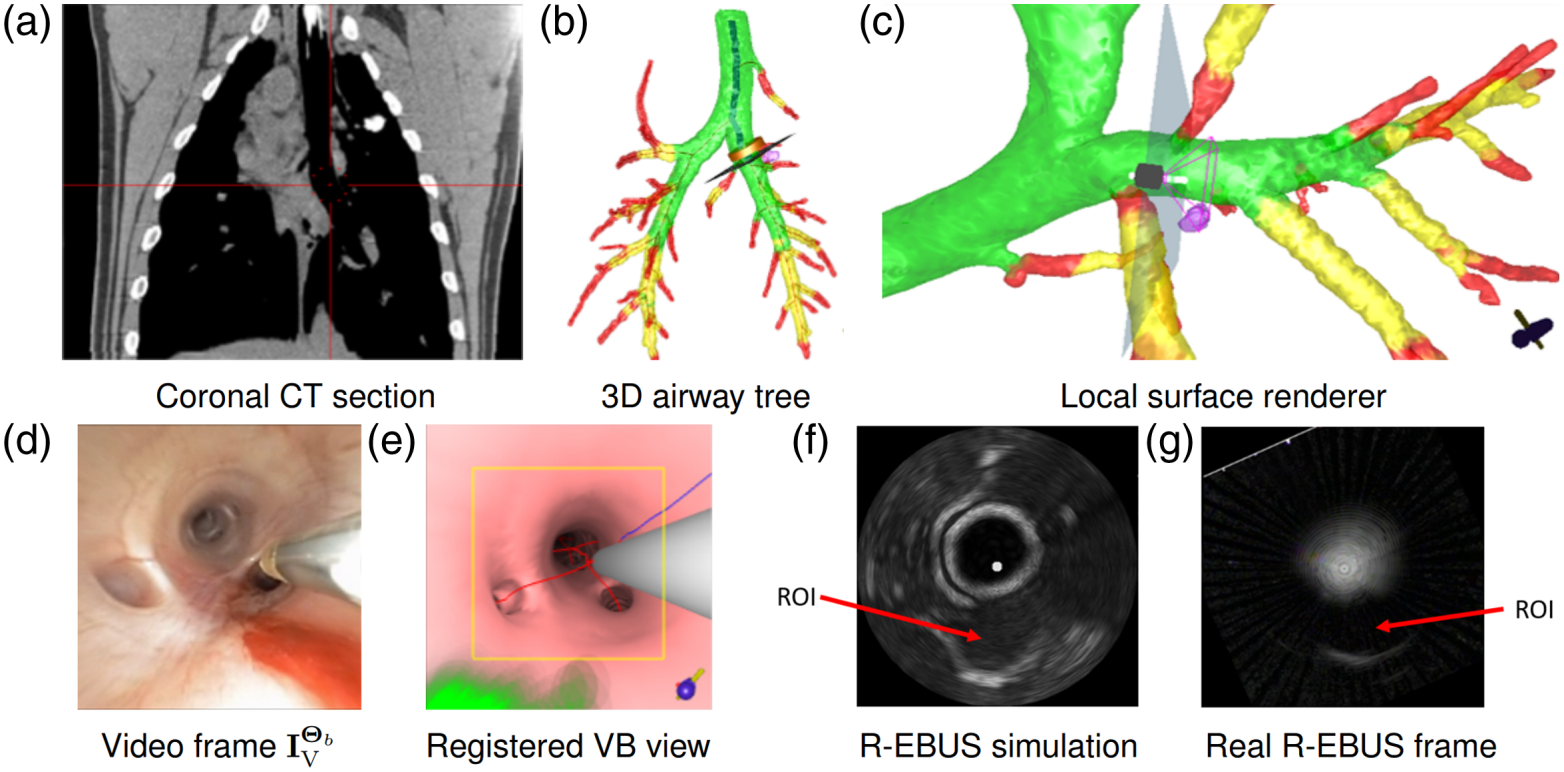
System example for animal study 2, ROI 1. All views are synchronized to the final registered site. Coronal CT section, 3D airway tree, and local surface renderer indicate the site, respectively, with red crosshairs, a yellow icon, and a scope tip icon. ROI is a purple region in panels (b)–(c). Coronal CT section and 3D airway tree in panels (a)–(b) are viewed back-to-front. Note that the dimensions of the R-EBUS images are 4×4  cm for all figures in this paper; Fig. 11(g) gives example scales. Panel (a) Coronal CT section, Panel (b) 3D airway tree, Panel (c) Local surface renderer, Panel (d) Video frame $I_{V}^{\Theta_{b}}$, Panel (e) Registered VB view, Panel (f) R-EBUS simulation Panel, and (g) Real R-EBUS frame.

Figure 10 gives a system example for pig #2. The figure demonstrates how our system’s supplemental graphical viewers can assist the physician in localizing a target ROI before performing R-EBUS scanning and final confirmation.

### 3.3 Human Studies

We next performed initial human studies to validate the method’s safety, feasibility, and functionality in a live clinical setting, following the standard lung cancer management workflow. The studies, which are ongoing, were performed under protocols approved by the Institutional Review Board of Penn State University, Hershey, PA (protocols 20,349 and 20,714, approval dates June 2023 and February 2024), with informed consent obtained for enrolled patients. For the studies, the physician used an Olympus BF-1TH190 bronchoscope and UM-S20-17S R-EBUS probe. All patients were held under general anesthesia during the procedure.

Our first lung cancer patient procedure served as a “dry run” attempt at employing the system live in the operating room following standard bronchoscopy procedures. The purpose of the study was to establish the system’s basic feasibility and safety in a real-time clinical environment. For this initial study, we de-emphasized initial navigation and chose shallow sites as test ROIs; in this way, the focus was on verifying the two-phase registration method for localizing an ROI using R-EBUS. (As stated earlier, it is already well accepted that assisted bronchoscopy systems effectively boost physician performance for bronchoscope navigation.)

**Fig. 11:**
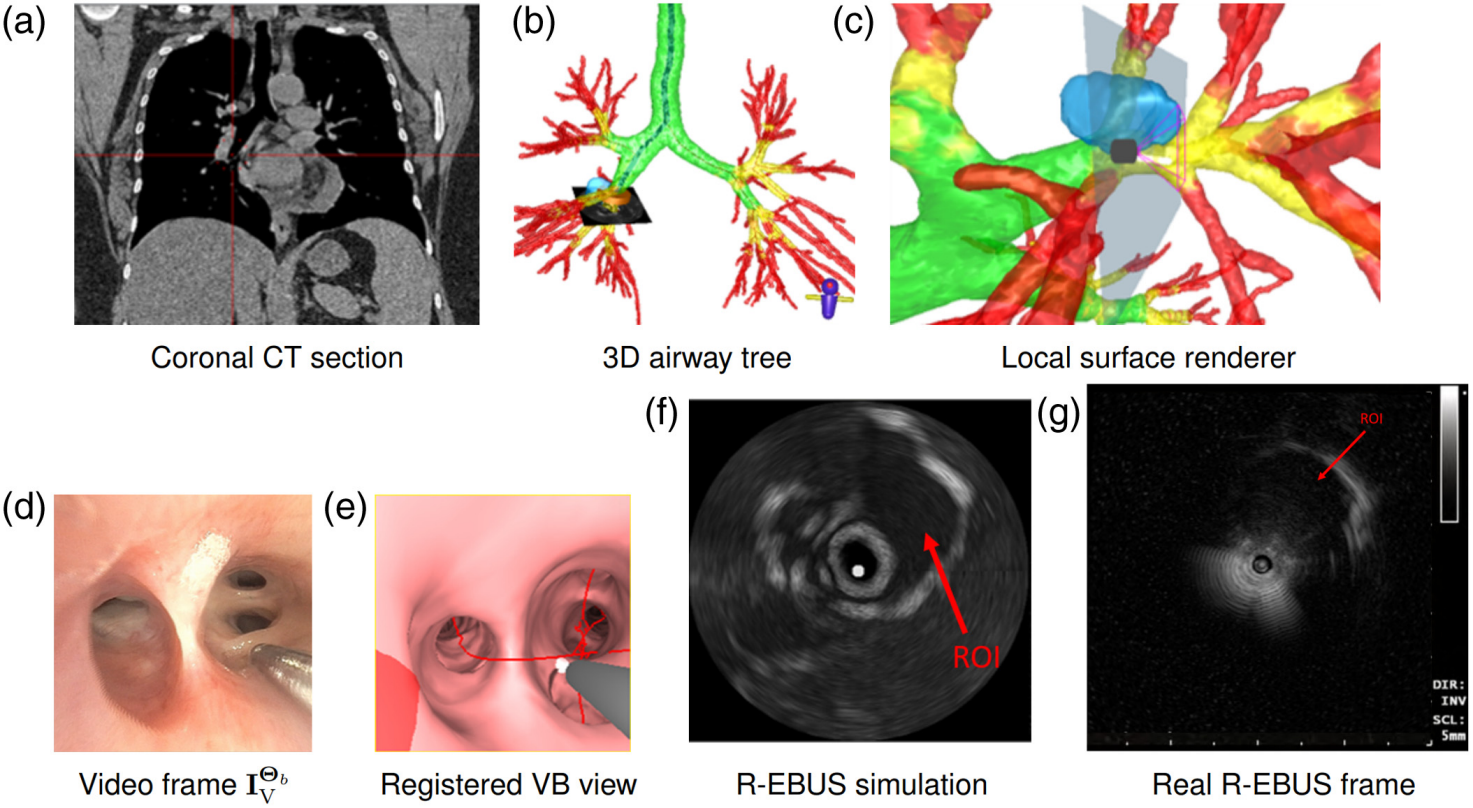
System example from the dry run study (patient 20349.3.98). All views synchronized to the final registered right lower lobe site. In panels (b)–(c), the blue regions denote the ROI, whereas icons indicate the location of the virtual bronchoscope. Planes perpendicular to the virtual bronchoscope denote the R-EBUS scan plane. Coronal CT section and 3D airway tree in panels (a)–(b) are viewed front-to-back. Note that panel (g) features the size axes (5 mm spacing between scale markers) and an intensity scale for reference; all R-EBUS images in this paper abide by these scales. Panel (a) Coronal CT section, Panel (b) 3D airway tree, Panel (c) Local surface renderer, Panel (d) Video frame $I_{V}^{\Theta_{b}}$, Panel (e) Registered VB view, Panel (f) R-EBUS simulation, and Panel (g) Real R-EBUS frame.

Figure 11 gives an example system view for this initial study for a site located in the right lower lobe. The displayed views give results after scanning the target lesion at the preplanned target site. These results clearly indicate that the two-phase registration method enabled the physician to perform an R-EBUS scan along the airway wall with confidence to immediately locate the extraluminal site. These results, coupled with the earlier phantom studies and controlled live animal studies, help establish the potential performance and live functionality of the method.

**Fig. 12:**
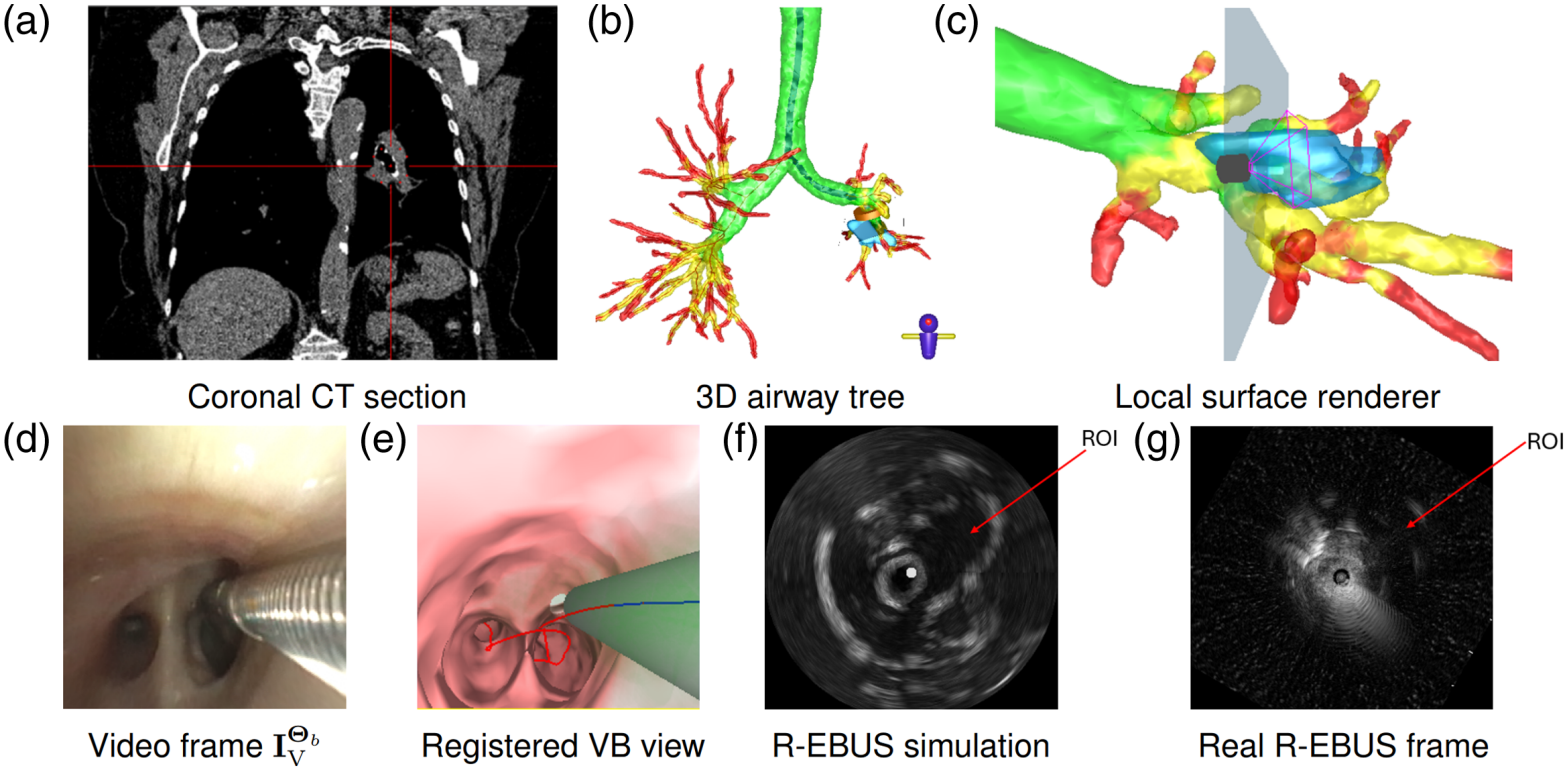
System example from the pilot study (patient 20714.06). The figures, organized as in Fig. 11, correspond to the system display upon reaching a generation-7 left lower lobe ROI. Panel (a) Coronal CT section, Panel (b) 3D airway tree, Panel (c) Local surface renderer, Panel (d) Video frame $I_{V}^{\Theta_{b}}$, Panel (e) Registered VB view, Panel (f) R-EBUS simulation, and Panel (g) Real R-EBUS frame.

We have recently embarked on a live pilot study of the system. Figure 12 gives a study example. For this substantially deeper generation-7 left lower site, the physician was immediately able to localize the target ROI with R-EBUS. Using the procedure time definitions of Table 2, the “Navigate + Insert” time was 109 s, and the “R-EBUS Register” time was 60 s for this ROI. In addition, the physician scanned the ROI for 6 s, giving a total live procedure time of 148 s.

## 4 Discussion and Conclusion

Because lung cancer continues to be the world’s leading cause of cancer death, considerable research has been focused on devising better methods for lung cancer management. This has led to the widespread introduction of CT-based early lung cancer screening,^65^ which in turn has driven a critical need for improved methods for disease diagnosis, staging, and treatment, based on ROIs identified on CT. The current state-of-the-art approach for performing these tasks, especially for extraluminal ROI sites situated beyond the central airways, involves using bronchoscopy in tandem with an assisted bronchoscopy guidance system.^2^^–^^4^^,^^6^

Although existing assisted bronchoscopy systems help the physician navigate the bronchoscope close to targeted ROIs, they offer no guidance for final ROI confirmation with R-EBUS, even though R-EBUS offers a safe, inexpensive, real-time approach for visualizing extraluminal ROIs. As a result, current bronchoscopy practice, even when using an assisted bronchoscopy system, generally requires resorting to a time-consuming radiation-intensive imaging modality, such as cone-beam CT or fluoroscopy, for final ROI confirmation. Thus, a critical need exists for a guidance method geared toward R-EBUS deployment and probe placement for final ROI confirmation. We addressed this need in existing assisted bronchoscopy systems by proposing a two-phase R-EBUS bronchoscopy registration method.

The method, tested within the context of an assisted bronchoscopy system, offers a potentially robust efficient guidance approach for ROI confirmation using R-EBUS, without the need for a radiation-based imaging modality. The two-phase registration process, comprising the alignment of both the bronchoscope and R-EBUS probe, enables precise synchronization between the virtual and real instruments. In addition, the method facilitates supplemental visual views that clearly indicate where to place the R-EBUS probe before scanning—this enables immediate visualization of the ROI, something that the supplemental imaging modalities do not explicitly provide. This allows a physician to perform an R-EBUS scan with greater confidence, thereby potentially reducing the likelihood of missing a target ROI and minimizing the variability in procedural outcomes associated with manual probe placement.

Validation of the method through a phantom study, animal experiments, and initial patient tests underscored its efficacy and safety under various conditions. The observed improvements in procedure time, along with the consistent accuracy in probe placement, highlight the method’s potential to streamline clinical workflow and enhance the overall efficiency of bronchoscopy procedures, particularly in the context of lung cancer management.

In 100% of our tests in phantom, animal, and human studies, the method enabled immediate visualization of a target lesion via R-EBUS. For the phantom studies, quantitative tests during probe placement indicated that mean accumulated position and direction errors (after registering both the bronchoscope and R-EBUS probe) were 1.94 mm (max = 4.93 mm) and 3.74 deg (max = 6.94 deg), respectively. The direction error is equivalent to a needle position error = 20  mm×sin(3.74°)=1.30  mm for a 20-mm biopsy needle. For the more challenging live animal studies, these errors were 2.81 mm (max = 4.62 mm) and 4.79 deg (max = 6.15 deg) [2.41 mm needle error], respectively. Given that the typical lesion has a long axis on the order of 10 mm, these errors point to the method’s potential precision for lesion confirmation.

We also observed typical procedure times to be <3  min per ROI, with roughly 80 s dedicated to final R-EBUS confirmation. As a comparison, recently reported procedure times in published studies using an assisted bronchoscopy system and a radiation-based imaging modality reported a mean procedure time per ROI >32  min.^16^^,^^18^ (Notably, both of these studies also drew on R-EBUS.) Regarding our method, the R-EBUS confirmation step took under 10 to 20 s in our studies, with a large majority of time taken by human interactions. Certain interactions could be streamlined or automated, but we did not do this in our prototyped system. As a further efficiency improvement, the two phases of registration required by our method could be parallelized. Regarding live clinical use, the initial procedures we performed in the operating room illustrated the practicality, safety, efficacy, and efficiency of the method during real-time clinical circumstances.

An alternative approach for tracking a surgical tool in video entails a simplified scenario using a projective tool model to estimate 3D pose.^66^^,^^67^ The approach, however, requires accurate tool outlines in the video, which can be difficult to obtain for the partially transparent borders of the R-EBUS probe. Hence, we chose a region-based alignment approach. Regarding this approach, Tjaden et al.^48^ demanded a template-matching strategy for pose estimation. Our scenario did not require this because the R-EBUS probe is always visible and emerging from the right side of the video frame. In addition, for our scenario, the probe is presegmented in the video frame to give a binary-valued image, which simplifies computations, unlike other applications needing true-color calculations.^48^

We note several limitations in our studies. First, our proposed method relies on a vision-based approach, whereby the bronchoscopic camera must be able to capture an image of the R-EBUS probe near the desired site. If the probe is not sufficiently clear, which could happen if the probe must be inserted deeply into a smaller peripheral airway, then registration can become problematic. In addition, the real R-EBUS probe can have ambiguity in its roll angle, arising from the probe’s rapid rotation during invocation. However, this can be compensated for by the known roll angle of the simulated EBUS view and subsequent probe registration. In addition, note that other working channel instruments, which our method can accommodate, are not subject to this ambiguity.

As a second limitation, although our presented studies do demonstrate the potential performance, feasibility, functionality, and safety of our method in the live clinical workflow, we have not shown true clinical efficacy. We are currently conducting a larger prospective clinical pilot study to better demonstrate feasibility and safety in the operating room. Nevertheless, a more detailed multi-center study, involving a commercial-grade-assisted bronchoscopy system, would be required to show true efficacy for a specific clinical application. In addition, although we believe our ground truth experiments for the phantom and animal studies offer plausible measures of the potential efficacy of our method, we also acknowledge that the ground truth could have some observer-based variability.

As a final limitation, our study has relied on devices that can only reach ROIs near an airway having a diameter ≥4  mm or approximately two-thirds of the potential airways.^12^^,^^27^^,^^68^ Accessing the remaining peripheral sites would require a bronchoscope having a diameter <4  mm, and the bronchoscope must also have a sufficiently large working channel to accommodate the R-EBUS probe. (The smallest current state-of-the-art Olympus bronchoscope with these characteristics has a distal diameter of 3.7 mm and a working channel diameter of 1.7 mm.) Device limitations, however, depend on the state of current technology and are not a limitation of our method per se. Furthermore, device technology continually improves, as evidenced by a newly introduced thinner endobronchial ultrasound bronchoscope capable of going deeper into the airway tree.^69^

As another comment, because the radiation-based imaging modalities, such as cone beam CT and fluoroscopy, have proven their value as reliable tools providing unique live imaging information, they will rightfully remain as valuable fail-safe devices for ROI confirmation in the surgical suite. As such, R-EBUS will not replace these modalities. R-EBUS, however, has shown its potential for cheaper, radiation-free ROI confirmation. Our method, which offers the advantages of a very short time window and more accurate direct placement of the R-EBUS probe, helps to not only make R-EBUS usable to a wider range of physicians but also encourage more effective use of the other imaging devices in conjunction with R-EBUS.

In conclusion, the two-phase registration method represents a significant advancement in image-guided bronchoscopy, offering a quick, low-cost, radiation-free approach to confirming extraluminal ROIs. As lung cancer diagnosis and treatment continue to evolve, the method has the potential to play a crucial role in improving patient outcomes, reducing procedural risks, and optimizing the use of bronchoscopy and other supplemental imaging modalities in clinical practice.

Last, although our studies focused on R-EBUS as the bronchoscope’s working channel instrument, nothing in our method depends on this instrument being an R-EBUS probe—all it requires is knowledge of the instrument’s shape. For example, to adapt the method to a biopsy needle, all that is required is a virtual mesh model tailored toward the needle’s shape, similar to Fig. 7. Thus, the method could be adapted to guide the use of many new cancer therapy/analysis working channel instruments devised for ablation therapy, optical coherence tomography, cryotherapy, and endomicroscopy.^3^^,^^23^^,^^32^^,^^33^ In this way, these devices could then see wider spread application. This is of considerable significance because as the patient population diagnosed with early-stage disease increases, the demand for accurate efficient therapy delivery becomes more critical.^19^^,^^23^

## 5 Appendix A: Probe Insertion Angle Experiment

This appendix presents a brief experiment to illustrate potential variations in R-EBUS probe insertion angle, a major influence on the efficacy of probe placement. It has been a common assumption that the mobility of an instrument inserted into a bronchoscope’s working channel, such as an R-EBUS probe, is significantly restricted by the bronchoscope’s tip design and by the working channel’s narrow diameter. This has led to the belief that it is straightforward to position an R-EBUS probe inserted into the bronchoscope’s working channel. Contrary to this belief, our findings imply that the inserted R-EBUS probe’s orientation angle can vary by over 30 deg.

**Fig. 13:**
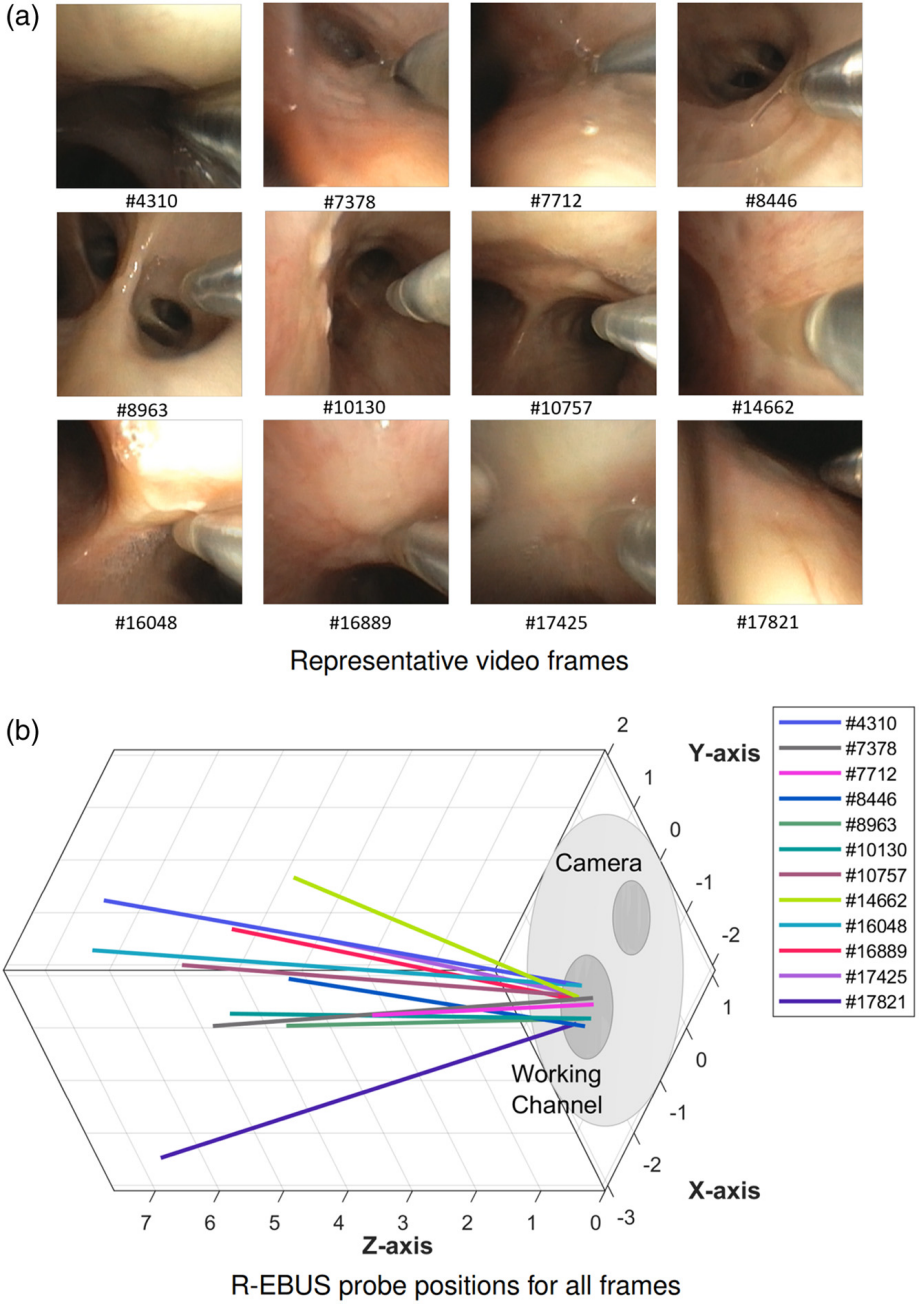
R-EBUS probe insertion positions at 12 different airway tree locations (patient case 21405.169). (a) Bronchoscopic video frames, indicated by frame number, showing the inserted R-EBUS probe at the twelve locations. (b) Orientations of the inserted R-EBUS probe represented as colored lines, relative to the bronchoscope’s tip, for the 12 positions of panel (a), where the X-Y plane at Z=0  mm denotes the bronchoscope tip. The units for the axes are in millimeters.

To demonstrate this, for 12 different bronchoscope positions within the airway tree, we measured the R-EBUS probe’s insertion angle after inserting the probe into the working channel. Figure 13(a) depicts bronchoscopic video frames captured at these twelve positions from a patient bronchoscopy. For the procedure, the physician used an Olympus BF-P190 bronchoscope (distal end diameter = 4.2 mm, working channel inner diameter = 2.0 mm) and an Olympus UM-S20-17S R-EBUS probe (1.4 mm diameter). For each frame, we determined the ground truth of the R-EBUS probe’s pose, relative to the bronchoscope camera by applying the methodology described in Sec. 2. We segmented the real probe in a video frame and interactively adjusted the virtual probe to find the best match to the real probe’s pose.

Figure 13(b) displays a plot of these probe positions, depicted as colored lines. In the plot, the bronchoscope’s distal end corresponds to the X-Y plane at Z=0  mm and conforms to the Olympus device’s known distal end geometry, with the working channel and bronchoscope camera situated as shown. A given line’s length represents the actual length that the probe extended from the tip for a given probe position (range: 3.7 to 7.7 mm). The plot clearly shows that the angular orientations of the probe positions varied significantly. This is likely due to the probe’s flexible design. Moreover, when the bronchoscope flexes, the probe inside the working channel also flexes. In addition, after the probe is pressed against the airway wall to make an R-EBUS scan, the probe’s position can change even more.

For this experiment, the angular differences observed between the various probe positions when compared pairwise varied over a range of 1.8 and 32.6 deg. The mean angular difference was 12.2  deg±6.5  deg (mean ± standard deviation). To understand how these differences can translate into pose errors, suppose the probe is extended 10 mm into the working channel. Then, an angular difference equal to 30 deg corresponds to a pose error equal to 5.18 mm. Considering that a typical lesion’s long axis is on the order of 10 mm, then a 10 mm insertion of the probe can easily result in missing a target lesion entirely, especially when accounting for physician skill differences.

## Data Availability

Data generated or analyzed during the study could become available from the corresponding author upon request.
